# A novel epitope-blocking ELISA for specific and sensitive detection of antibodies against H5-subtype influenza virus hemagglutinin

**DOI:** 10.1186/s12985-021-01564-6

**Published:** 2021-04-30

**Authors:** Violetta Sączyńska, Katarzyna Florys-Jankowska, Anna Porębska, Violetta Cecuda-Adamczewska

**Affiliations:** ŁUKASIEWICZ Research Network - Industrial Chemistry Institute, Rydygiera 8 Street, 01-793 Warsaw, Poland

**Keywords:** Avian influenza viruses, Hemagglutinin, Monoclonal antibodies, Antibody detection, Blocking ELISA, H5, Serology

## Abstract

**Background:**

H5-subtype highly pathogenic (HP) avian influenza viruses (AIVs) cause high mortality in domestic birds and sporadic infections in humans with a frequently fatal outcome, while H5N1 viruses have pandemic potential. Due to veterinary and public health significance, these HPAIVs, as well as low pathogenicity (LP) H5-subtype AIVs having a propensity to mutate into HP viruses, are under epidemiologic surveillance and must be reported to the World Organization for Animal Health (OIE). Our previous work provided a unique panel of 6 different monoclonal antibodies (mAbs) against H5 hemagglutinin (HA), which meets the demand for high-specificity tools for monitoring AIV infection and vaccination in poultry. In this study, we selected one of these mAbs to develop an epitope-blocking (EB) ELISA for detecting H5 subtype-specific antibodies in chicken sera (H5 EB-ELISA).

**Methods:**

In the H5 EB-ELISA, H5 HA protein produced in a baculovirus-expression vector system was employed as a coating antigen, and the G-7-27-18 mAb was employed as a blocking antibody. The performance characteristics of the assay were evaluated by testing 358 sera from nonimmunized chickens and chickens immunized with AIVs of the H1–H16 subtypes or recombinant H5 HA antigen to obtain the reference and experimental antisera, respectively. The samples were classified as anti-H5 HA positive or negative based on the results of the hemagglutination inhibition (HI) assay, the gold standard in subtype-specific serodiagnosis.

**Results:**

The H5 EB-ELISA correctly discriminated between the anti-H5 HA negative sera, including those against the non-H5 subtype AIVs, and sera positive for antibodies against the various-origin H5 HAs. Preliminary validation showed 100% analytical and 97.6% diagnostic specificities of the assay and 98.0% and 99.1% diagnostic sensitivities when applied to detect the anti-H5 HA antibodies in the reference and experimental antisera, respectively.

**Conclusions:**

The H5 EB-ELISA performed well in terms of diagnostic estimates. Thus, further optimization and validation work using a larger set of chicken sera and receiver operating characteristic (ROC) analysis are warranted. Moreover, the present assay provides a valuable basis for developing multispecies screening tests for birds or diagnostic tests for humans.

**Supplementary Information:**

The online version contains supplementary material available at 10.1186/s12985-021-01564-6.

## Background

Influenza viruses (IVs) belong to the *Orthomyxoviridae* family, which consists of seven genera [1]. Strains of the most epidemiologically relevant *influenza A virus* species of the *Alphainfluenzavirus* genus are enveloped negative-sense single-strand RNA viruses with a segmented genome [2]. Eight RNA segments encode RNA polymerase subunits, nucleoprotein (NP), matrix protein (M1) and membrane protein (M2), nonstructural protein (NS1) and nuclear export protein (NEP), and surface glycoproteins, hemagglutinin (HA) and neuraminidase (NA). Influenza A viruses have antigenically related NP and M1 proteins [3]. They are classified into subtypes on the basis of their variable HA and NA antigens. A total of 16 HA subtypes (H1–H16) and 9 NA subtypes (N1–N9) have been identified in wild aquatic birds, the major reservoir of influenza A viruses. Avian influenza viruses (AIVs) are usually nonpathogenic in their natural waterfowl hosts, and most of them are of low pathogenicity (LP) for domestic birds. However, the H5- and H7-subtype AIVs may become highly pathogenic (HP) by mutation once introduced into the poultry population.

HPAIVs caused sporadic epizootics in domestic poultry until 1996 [4]. Since then, the H5N1 HPAIV has become enzootic in poultry within several countries. Concomitant with the initial circulation and spread of the H5N1 viruses, HA genes diversified into multiple genetic lineages, termed clades [5]. In 2008, substantial reassortment events began and resulted in the creation of a range of H5Nx reassortants, such as H5N2, H5N6 and H5N8 HPAIVs, that have acquired novel NA proteins [6]. Emerging outbreaks of disease evoked by H5-subtype HPAIVs are accompanied by high virulence and mortality among domestic birds, which leads to enormous economic losses for the poultry industry [5, 6]. Moreover, the H5N1 and H5N6 strains of the virus cause sporadic infections in humans [6], and fatal cases of human infection with H5N1 viruses are still noted [7]. The important issue is the pandemic potential of H5N1 HPAIVs. Due to the threat to animal and human health and life, H5-subtype HPAIVs are under epidemiologic surveillance [3]. LPAIVs of the H5 subtype are also a serious concern, as they have the potential to mutate into HP viruses. Accordingly, the occurrence of both the HP and LP H5Nx viruses must be reported to the World Organization for Animal Health (OIE). Therefore, all of the H5-subtype viruses are classified as notifiable AIVs (NAIVs).

Research on the evolution, spreading and occurrence of novel strains of H5Nx AIVs requires specific and reliable methods for virus and antibody detection, subtype identification and pathotype classification [4] that are prescribed by the OIE [3]. In early influenza diagnostics, influenza virus is detected primarily using the real-time reverse transcriptase polymerase chain reaction (rRT-PCR) assay, virus isolation and antigen immunoassays. Subsequently, the subtype of the virus may be identified with rRT-PCR or sequencing the HA and NA genes and pathotype of the H5 or H7 subtype virus by in vivo chicken testing and determining the sequence of the HA proteolytic cleavage site. To assess prior exposure to AIVs, serological diagnosis of influenza is performed using the two-step procedure. The detection of antibodies to any AIV is followed by identification of the virus subtype. Detecting the antibodies to the group antigens of influenza A viruses, the NP and/or M1 proteins, is routinely accomplished using the agar gel immunodiffusion (AGID) test and enzyme-linked immunosorbent assay (ELISA). For determination of the HA and NA subtypes, hemagglutination inhibition (HI) and neuraminidase inhibition (NI) tests are recommended, respectively. The general assessment of the current tests for most AIV testing is that they are adequate, while some modifications, updates or additional tests would be beneficial [4]. The element of AIV diagnostics that is the most in need of improvement is in determining the HA and NA subtype specificity of antibodies to AIV.

In subtype-specific serodiagnosis, the HI test is considered to be the ‘gold standard’ [8]. Its accuracy can be near perfect, however, only when the reference viral antigen is close enough to the virus isolate to be tested. To avoid the risk of false negative results, a panel of different antigens should be used in the initial analyses. The issues that need to be considered while performing the HI test are also nonspecific hemagglutination and nonspecific inhibition of hemagglutination [3]. They may be circumvented by sample pretreatment and using at least two antigens of the same HA subtype but with different NA subtypes. The assays require several control samples. Thus, the HI test is demanding with respect to the protocol, reagents and interpretation. Moreover, it is labor-intensive, time-consuming and difficult to automate, which restricts its use in large-scale sero-surveillance studies. Due to substantial limitations of the HI assay, subtype-specific ELISAs in competitive and blocking formats (cELISA and bELISA, respectively) have been developed as high-throughput screening tests [8]. Their accuracy has in most cases been evaluated in reference to the HI test. Currently, some subtype-specific ELISA kits are becoming available such as kits for antibodies to H5 and H7 HAs and N1 NA [3]. In addition to serodiagnosis, such kits may be successfully used in AIV vaccination programs for differentiating infected animals from vaccinated animals as so-called DIVA tests. In cELISAs and bELISAs, subtype-specific monoclonal antibodies (mAbs) are employed as essential reagents.

Diversity of the H5-subtype AIVs and continual variability of HA antigens are objective difficulties and simultaneously a challenge in serodiagnosis and surveillance of these viruses. In particular, the development of diagnostically valuable cELISA or bELISA tests requires the use of mAbs recognizing intrasubtype-conserved epitopes in the HA antigen. This requirement is met by the highly and broad-range specific mAbs against H5 HAs, which were produced and differentiated by us according to the procedures presented previously, together with the results from immunoreactivity studies [9]. In this work, the diagnostic value of the newly generated mAbs was positively verified by using one of the 6 antibody clones, the G-7-27-18 mAb, in epitope-blocking (EB) ELISA for detecting H5 subtype-specific antibodies in chicken sera. Here, the current characteristics of the developed test, referred to as H5 EB-ELISA, are shown, and its future prospects are discussed.

## Methods

### MAb production and selection

MAbs against H5 HA were generated according to the procedure described previously [9]. Briefly, five 6-week-old female BALB/c mice were immunized with purified recombinant H5 HA protein produced in a mammalian expression system (Immune Technology Corp., New York, NY, USA). Details of the protein, referred to as rH5-mammalian, are provided in Additional file 1: Table S1. The first 10-µg dose of immunogen, emulsified with an equal volume of complete Freund’s adjuvant (Sigma-Aldrich, St. Louis, MO, USA), was administered subcutaneously. The booster doses, each containing 10 μg of rH5-mammalian in PBS, were given twice by intraperitoneal injection and once intravenously. Three days after the last immunization, splenocytes were isolated and fused with mouse myeloma of the SP2/0 cell line (ATCC, Rockville, MD, USA). The fused hybrid cells were cultured in RPMI-1640 medium containing FBS, l-glutamine, sodium pyruvate, and antibiotics, with hypoxanthine, aminopterin and thymidine (HAT) as the selecting agents. The hybridomas were subcloned by the limited dilution method. The resulting hybridoma cell lines were grown in RPMI-1640 medium with the same supplements as the selection culture medium except for HAT. The reagents used for fusion and hybridoma culture were purchased from Sigma-Aldrich.

The hybridoma culture supernatants were screened for the presence of IgG antibodies against H5 HA using ELISAs that targeted the various H5 HA antigens. They included proteins from a mammalian expression system (Immune Technology Corp.) and a baculovirus-expression vector system (Oxford Expression Technologies Ltd., Oxford, England, UK). In addition, inactivated, H5-subtype AIVs (x-OvO Ltd., Dunfermline, Scotland, UK), certified by Istituto Zooprofilattico Sperimentale delle Venezie (IZSVe; Legnaro, Padova, Italy), were used. The sequences of the HA antigens employed in the positive hybridoma selection originated from the highly divergent H5-subtype influenza viruses. The selected mAbs, for a total of 7 clones, were purified from the hybridoma culture supernatants using a HiTrap Protein G HP column (GE Healthcare, Uppsala, Sweden).

### Analyses of selected mAbs

The finally selected, affinity-purified mAbs, denoted G-1-31-22, G-2-14-10, G-5-32-5, G-6-42-42, G-6-42-71, G-7-24-17 and G-7-27-18, were characterized by isotyping, immunoreactivity studies and peptide mapping, as described previously [9]. Isotyping was performed using a commercial kit: ‘Mouse Monoclonal Antibody Isotyping Reagents’ (ISO-2; Sigma-Aldrich). The reactivity range of the selected mAbs was determined using ELISAs against well-characterized recombinant H5 HA proteins produced in mammalian or insect cells (Immune Technology Corp., Oxford Expression Technologies Ltd., respectively) and inactivated, H5-subtype AIVs (x-OvO Ltd.) certified by IZSVe (Legnaro, Padova, Italy). As a result, the H5 HA antigens varied in form (recombinant proteins or influenza viruses), length (HA fragments or full-length HAs), conformation (properly folded or misfolded), oligomerization state (monomeric or at least partly oligomeric) and glycosylation pattern (mammalian or insect). Conformational H5 HA antigens used in the specificity analyses included the sequences of 12 strains of HP or LP and H5-subtype AIVs. Consistently, they had diverse amino acid sequences, especially within the subtype-determining HA1 subunit, which was confirmed by a homology search against the immunogen (BLAST program, NCBI). To ascertain the cross-reactivity of the obtained mAbs, 21 strains of AIVs (x-OvO Ltd.) certified by IZSVe (Legnaro, Padova, Italy) were used for testing. They represented the H1–H4 and H6–H16 subtypes of influenza viruses. Apart from determination of the specificity range, the mAb examination by ELISAs comprised the binding capability assessment and immunoreactivity profiling. Peptide mapping was performed for the Fab and Fc antibody fragments digested with trypsin using a matrix-assisted laser desorption ionization time of flight (MALDI-TOF/TOF) mass spectrometer (Applied Biosystems, Waltham, MA, USA). On this basis, the profiles of the tryptic peptide maps of individual antibody clones were defined.

The newly generated antibodies against H5 HA were also subjected to the HI test with H5N2 and H5N3 LPAIVs (x-OvO Ltd.) as the reference antigens. Among the H5N1, H5N2, H5N3 and H5N9 AIVs previously employed in ELISAs for antibody specificity, the H5N2 virus had the lowest and the H5N3 virus the highest homology to the HA1 subunit of immunogen used in mAb production (Additional file 1: Table S6). In the test, the reference anti-H5N2, anti-H5N3 and anti-H7N7 LPAIV antisera (x-OvO Ltd.) as well as commercial mAbs against H5 HA (Acris Antibodies GmbH, Herford, Germany; Pierce/Thermo Fisher Scientific, Waltham, MA, USA) served as positive or negative controls (Additional file 2: Tables S1 and S2). The test was performed using erythrocytes of specific pathogen-free (SPF) chickens obtained from the Department of Poultry Diseases, National Veterinary Research Institute (DPD NVRI, Puławy, Poland). Details are provided in Additional file 2.

### Serum samples

A panel of 358 chicken serum samples comprised the anti-LPAIV reference antisera, experimental antisera specific for H5 HA and anti-H5 HA negative sera. HA antigens used to obtain the antiserum samples had sequences derived from a total of 26 virus strains.

Reference antisera, certified by IZSVe (Legnaro, Padova, Italy), were purchased from x-OvO Ltd. They were produced in the SPF chickens inoculated with 25 strains of inactivated LPAIVs representing the HA subtypes from H1 to H16. The H5-subtype viruses used for chicken immunization were H5N1, H5N2, H5N3 and H5N9 LPAIVs. In this work, 1, 2 or 3 batches of each anti-AIV antiserum were used. Consistently, the set of reference antisera comprised 31 samples. The presence of subtype-specific antibodies in these samples was confirmed by HI assays with homologous virus strains. The values of HI titers were included in the product certificates.

Experimental antisera were from previously presented efficacy trials for the HA protein of the H5N1 HPAIV produced in *Escherichia coli* [10]. The vaccine antigen, referred to as rH5-*E. coli*, was refolded and chromatographically purified from inclusion bodies. Immunization studies were performed in the Rossa 1 line of layer chickens. The chickens were purchased from a commercial breeder on the day of hatching and maintained at an experimental poultry house with wheat straw bedding. Vaccines dedicated for commercial flocks were not administered. Eight groups of 3-week-old layers were vaccinated twice with 25 µg, 15 µg, 10 µg, or 5 µg of rH5-*E. coli* and aluminum hydroxide (alum) adjuvant. In this work, a total of 115 samples collected 1 and/or 2 weeks after the boost were used.

The anti-H5 HA negative sera were from control groups in the immunization studies with rH5-*E. coli*, including those reported previously [10]. The studies were performed under semifield conditions, described above, using the commercial layers and broilers—namely, the Rossa 1 and Ross 308 lines, respectively. Samples were collected from the nonimmunized chickens at different time points of the experiments. Thus, the obtained panel of 191 samples negative for H5 HA was completed with 18 sera from SPF layers, the White Leghorn line (DPD NVRI, Puławy, Poland), and 1 batch of normal chicken serum (Abcam, Cambridge, England, UK).

Serum samples and their applications in the development and evaluation of EB-ELISA are described in Table 1. Influenza virus strains used to obtain reference antisera against H5 and non-H5 subtype AIVs are listed in Additional file 1: Tables S2 and S3, respectively, together with other details of serum samples. Supplementary data on the vaccine antigen, rH5-*E. coli*, and the anti-H5 HA experimental antisera are provided in Additional file 1: Tables S1 and S4, respectively.

**Table 1:** Sera used for H5 EB-ELISA development and preliminary validation

Chickens	Immunogen	Serum description	Samples (N)	HI titer	Origin
Anti-H5 HA positive (1): reference antisera against LPAIVs of the H5 subtype					
Used in evaluation of diagnostic sensitivity 1 (Dse 1)					
SPF	H5N1 AIV, inactivated	One batch (#1)	1	1:512 with H5N1 AIV according to the certificate	x-OvO Ltd.^1a^
SPF	H5N2 AIV, inactivated	Three batches (#1, #2 and #3)	3	1:256 or 1:512 with H5N2 AIV according to the certificates	x-OvO Ltd.^1a^
SPF	H5N3 AIV, inactivated	Three batches (#1, #2 and #3)	3	1:512 with H5N3 AIV according to the certificates	x-OvO Ltd.^1a^
SPF	H5N9 AIV, inactivated	Two batches (#1 and #2)	2	1:512 or 1:256 with H5N9 AIV according to the certificates	x-OvO Ltd.^1a^
Positive controls (PC) used for repeatability determination					
SPF	H5N2 AIV, inactivated	Batch #3 (weak PC)	1	1:512 with H5N2 AIV according to the certificate	x-OvO Ltd.^1a^
SPF	H5N3 AIV, inactivated	Batch #1 (strong PC)	1	1:512 with H5N3 AIV according to the certificate	x-OvO Ltd.^1a^
Anti-H5 HA positive (2): experimental antisera against HA from H5N1 HPAIV					
Used in evaluation of diagnostic sensitivity 2 (Dse 2)					
Commercial layers, Rossa 1 line	rH5-*E. coli* adjuvanted with alum	From 69 chickens at 8, 9, 10 or 11 weeks of age; 1 or 2 sampling time points per chicken	115 (115)	1:8–1:512 with H5N2 AIV, determined at IBA	IBA^2a^
Anti-H5 HA negative (1): sera of various origin					
Used in determination of cutoff value and diagnostic specificity (Dsp)					
SPF layers, White Leghorn line	None	From 10 chickens at 9 or 11 weeks of age; 1 or 2 sampling time points per chicken	18	Negative AI status	DPD NVRI^3^
Commercial layers, Rossa 1 line	None	From 30 chickens at 7, 8, 9, 10 or 11 weeks of age; 3–5 sampling time points per chicken	130 (63)	< 1:8 with H5N2 AIV, determined at IBA	IBA^2b^
Commercial broilers, Ross 308 line	None	From 42 chickens at 3, 5, 5 ½, 6 or 7 weeks of age; 1–4 sampling time points per chicken	61 (33)	< 1:8 with H5N2 AIV, determined at IBA	IBA^2b^
Negative control (NC) used for repeatability determination					
Different strains and sex	None	Normal chicken serum, 1 batch (NC)	1	< 1:8 with H5N2 AIV, determined at IBA	Abcam^4^
Anti-H5 HA negative (2): reference antisera against LPAIVs of the non-H5 subtypes					
Used in evaluation of analytical specificity (Asp)					
SPF	AIVs: H1–H4, H6–H12 and H14–H16 inactivated	One batch of each antiserum	20	1:128–1:2048 with homologous AIVs according to the certificates	x-OvO Ltd.^1b^
SPF	H13N6 AIV, inactivated	Two batches (#1 and #2)	2	1:128 or 1:1024 with H13N6 AIV according to the certificates	x-OvO Ltd.^1b^

^1^Certified by Istituto Zooprofilattico Sperimentale delle Venezie (IZSVe; Legnaro, Padova, Italy) and purchased from x-OvO Ltd. (Dunfermline, Scotland, UK). Details of reference antisera against (a) H5 and (b) non-H5 subtype AIVs are provided in Additional file 1: Tables S2 and S3, respectively

^2^Collected in (a) the test vaccine and (b) control chicken groups during immunization studies with H5 HA protein produced in bacteria (rH5-*E. coli*) at the Institute of Biotechnology and Antibiotics (IBA; Warsaw, Poland). The number of samples described previously with HI titers [10] are provided in brackets. Details of the vaccine antigen and experimental antisera against H5 HA are presented in Additional file 1: Tables S1 and S4, respectively

^3^Obtained from the Department of Poultry Diseases, National Veterinary Research Institute (DPD NVRI; Puławy, Poland)

^4^(Cambridge, England, UK), Cat. No. b7477

### Hemagglutinin inhibition (HI) assay

HI activity was determined for sera from commercial chickens immunized and nonimmunized with rH5-*E. coli*, as indicated in Table 1. The reference viral antigen and antisera used in the HI assay were from x-OvO Ltd. Details are provided in Additional file 1: Table S5. Normal chicken serum (Table 1) was analyzed using reference reagents from the Veterinary Laboratories Agency (New Haw, England, UK).

The HI assay was performed according to the OIE Manual of Diagnostic Tests and Vaccines for Terrestrial Animals [11] following the previously described procedure [10]. Briefly, the sera were analyzed with the heterologous A/turk/Italy/80(H5N2) LPAIV strain at an HI unit (HIU) of 1:8 using SPF chicken erythrocytes (DPD NVRI, Puławy, Poland). Each assay included control erythrocytes, the antiserum against H5N2 LPAIV as a positive control and anti-H7N4 and/or anti-H7N7 LPAIV antisera as negative controls. The HI titer was defined as the reciprocal of the highest dilution of serum that caused an inhibition of hemagglutination activity with 4 hemagglutination units (HAU) of the inactivated antigen. In this study, serum HI titers equal to or greater than 1:8 were considered positive. On this basis, each serum sample was scored as positive or negative for H5 subtype-specific antibodies. The results of the HI assay for 115 antisera collected in the layers immunized with rH5-*E. coli* and 96 sera from the nonimmunized layers and broilers were adapted from our previous paper [10].

### Epitope-blocking ELISA (EB-ELISA)

EB-ELISA was performed using the purified recombinant H5 HA protein (aa 17–530, ΔRRRKKR, 6 × His) produced in a baculovirus-expression vector system (BEVS; Oxford Expression Technologies Ltd.). Details of the protein, referred to as rH5-BEVS, are provided in Additional file 1: Table S1. The MediSorp plates (Nunc, Roskilde, Denmark) were coated by overnight incubation at 2–8 °C with 50 μL/well of rH5-BEVS at a concentration of 0.5 μg/mL in PBS. The coated plates were washed three times with 300 μL/well of PBS containing 0.05% Tween 20 (PBST; pH 7.4) and then incubated with 200 μL/well of Protein-Free T20 (PBS) Blocking Buffer (Pierce/Thermo Fisher Scientific) for 1 h at room temperature (23 ± 2 °C). After washing two times with 350 μL/well of PBST, incubation buffer (1% BSA in PBS) was applied to the plates at 50 μL or 100 μL per well. Sera at 50-μL volumes were added to the wells with 50 μL of incubation buffer, resulting in a twofold sample dilution. The wells with 100 μL of incubation buffer were left without the serum addition to provide a control of maximum mAb binding to rH5-BEVS (mAb control). Each assay for the test sera, listed in Table 1, was performed in the presence of other control samples. The anti-H5N2 and anti-H5N3 LPAIV antisera were the weak and strong positive controls, respectively (Table 1, Additional file 1: Table S2), while the normal chicken serum (Table 1) was a negative control. All sera were analyzed in duplicate. The plates with test and control samples were incubated for 1 h at 37 °C with shaking at 150 rpm and subsequently washed three times with 300 μL/well of PBST. Next, 50 μL/well of G-7-27-18 mAb, diluted to 1 μg/mL in Antibody Stabilizer PBS (CANDOR Bioscience GmbH, Wangen, Germany), was applied to the plates, which were then incubated again for 1 h at 37 °C with shaking at 150 rpm and washed three times with 300 μL/well of PBST afterwards. Antigen-bound mAbs were detected using HRP-labeled, anti-mouse IgG (γ-chain specific) antibodies (Sigma-Aldrich). The plates were incubated with 50 μL/well of anti-mouse antibodies, diluted 1:3,500 in HRP-Protector (CANDOR Bioscience GmbH), for 1 h at 37 °C with shaking at 150 rpm and then washed three times with 300 μL/well of PBST. The reactions were developed with 50 μL/well of TMB (Sigma-Aldrich) at room temperature (25 ± 0.1 °C) in the dark for 15 min and subsequently stopped by adding 50 μL/well of 0.5 M H_2_SO_4_.

The optical density (OD) was measured at 450 nm (OD_450_) using a Synergy 2 multidetection microplate reader (BioTek Instruments Inc., Winooski, VT, USA). For each test and control sample, the mean OD_450_ value was calculated. The reduction of mAb binding to rH5-BEVS caused by individual specimens was expressed as inhibition percentage calculated using the formula: inhibition percentage = 100 − [100 × (OD_450_ of specimen/OD_450_ of mAb control)].

## Results

### Production and characteristics of mAbs

MAbs against H5 HA were produced with hybridoma technology as described previously [9]. For mouse immunization, the purified, ectodomain-based H5 HA protein (aa 17–530, ΔRRRKKR, 6 × His) from a mammalian expression system was used (Additional file 1: Table S1). The sequence of the protein, named rH5-mammalian, was derived from the A/Bar-headed Goose/Qinghai/12/05(H5N1) strain of HPAIV. As shown in our previous papers [9, 12], the antigen had characteristics of viral HA. The procedure for obtaining the anti-H5 HA antibodies consisted of spleen/myeloma fusion, screening of the resulting hybridomas and subcloning. In the positive hybridoma selection, the well-characterized recombinant H5 HA proteins were employed in addition to the H5-subtype influenza viruses. This strategy led to the selection of 7 hybridoma cell lines and their respective antibody clones. The affinity-purified antibodies, all of IgG1 isotype, had the same range of immunoreactivity as determined by ELISA. To differentiate antibody clones, immunoreactivity profiling and peptide mapping of antibody fragments were performed [9]. In this way, 6 different anti-H5 HA antibody clones, denoted G-1-31-22, G-2-14-10, G-5-32-5, G-6-42-42, G-7-24-17, and G-7-27-18, were identified.

According to previously published results [9], the newly established mAbs specifically recognize epitopes in the properly folded HA1 subunit of HAs from multiple strains of the H5-subtype influenza viruses representing both the HP and LP phenotypes. Among these were the H5N3 (1 strain), H5N9 (1 strain), and H5N2 (2 strains) viruses and the H5N1 viruses (8 strains) belonging to 5 clades. The HA1 subunits of these antigens shared 88 to 99% of their amino acid sequence identities with the HA1 subunit of the immunogen. Moreover, none of the 6 antibody clones cross-reacted with AIVs of the H1–H4 and H6–H16 subtypes. To complete the analysis of their characteristics, the G-1-31-22, G-2-14-10, G-5-32-5, G-6-42-42, G-7-24-17 and G-7-27-18 mAbs were examined in the HI assay. The test was performed following the protocol outlined in Additional file 2. As presented in Additional file 2: Tables S3 and S4, none of the antibody clones inhibited hemagglutination by H5N2 and H5N3 LPAIVs. To summarize the results presented in the previous [9] and this paper, we have established a unique panel of 6 different conformation-sensitive antibody clones, each of which is highly and broad-range specific against HAs of the H5-subtype, HP and LP AIVs and lacks HI activity. These properties make the antibodies useful analytical tools, particularly for diagnosing infections with H5Nx influenza viruses and the DIVA strategy in AIV vaccination campaigns.

### Development of an epitope-blocking (EB) ELISA

To verify the diagnostic value of the newly generated mAbs, we developed H5 EB-ELISA, the epitope-blocking ELISA for the detection of anti-H5 HA antibodies. The G-7-27-18 mAb was chosen to be a blocking antibody since among the 6 antibody clones, it reacted most uniformly with various H5 HA antigens, as revealed in the immunoreactivity profiles [9]. To avoid steric hindrance by the NA protein, the H5 HA protein was employed as the coating antigen instead of the commonly used inactivated viral antigen. This was the purified, ectodomain-based H5 HA protein (aa 17–530, ΔRRRKKR, 6 × His) from a baculovirus-expression vector system (Additional file 1: Table S1). The sequence of the protein, referred to as rH5-BEVS, originated from the A/swan/Poland/305-135V08/2006(H5N1) strain of HPAIV. Consistent with previously published results [9, 12], the antigen was correctly folded and existed in part as a functional oligomer. To detect the complex of rH5-BEVS and G-7-27-18 mAb, anti-mouse IgG (γ-chain specific) antibodies labeled with HRP and TMB as the HRP substrate were used. Under the principle EB-ELISA, the assay gives a positive result when antibodies to H5 HA in test sera block binding of mAb to the target epitope in the coating antigen and thus the color development. Consistently, the ELISA OD values are inversely proportional to the number of epitope-specific antibodies present in the samples.

The H5 EB-ELISA was optimized. The G-7-27-18 mAb was titrated against rH5-BEVS coated on the well surfaces with varied hydrophilicity (Additional file 3: Fig. S1). In this way, the MediSorp plates were selected to provide the best antigen binding and epitope presentation to the blocking antibody. The plates coated with rH5-BEVS were exploited in testing a panel of samples representing the anti-H5 HA positive and negative sera listed in Table 1. Samples were analyzed at a twofold dilution under different assay conditions. As a result, the blocking and dilution buffers were specified, and the period and temperature of plate incubation at subsequent stages of the assay procedure were established. The optimum concentrations of rH5-BEVS and G-7-27-18 mAb as well as dilution of HRP-labeled, anti-mouse IgG antibodies were determined by titrations. The relevant data are provided in Additional file 3: Figs. S2-S5.

### Diagnostic performance of EB-ELISA

The performance characteristics of the H5 EB-ELISA were evaluated by testing the sera of chickens immunized and not immunized against influenza viruses (Table 1, Additional file 1: Tables S2-S4). The samples were from different-age SPF and non-SPF chickens, representing the two main types of chicken breeds, layer and broiler. They were classified as anti-H5 HA positive or negative based on the results of the HI assay with H5-subtype AIVs. In this study, sera were considered positive when their HI titers were equal to or greater than 1:8. Categories of test samples and their application in the H5 EB-ELISA assessment are provided in Table 1.

The anti-H5 HA-positive samples comprised the reference and experimental antisera (Table 1). They were obtained by immunization of SPF chickens with H5-subtype LPAIVs and commercial chickens with recombinant H5 HA protein. The reference antisera were raised against H5N1, H5N2, H5N3 and H5N9 LPAIVs and had HI titers of 1:256 or 1:512 with homologous virus strains (Additional file 1: Table S2). Experimental antisera originated from previously described semifield trials for vaccine efficacy of the ectodomain-based HA protein produced in *Escherichia coli* [10]. The sequence of the protein, referred to as rH5-*E. coli*, was derived from the H5N1 A/swan/Poland 305-135V08-2006 strain of HPAIV, which is the same strain as rH5-BEVS (Additional file 1: Table S1). As shown previously [12], refolded and purified antigen displayed native HA characteristics. Commercial layer chickens were vaccinated twice with different doses of alum-adjuvanted rH5-*E. coli* [10]. For analyses, postvaccination sera with HI titers from 1:8 to 1:512 against heterologous H5N2 LPAIV were selected (Additional file 1: Table S4).

The panel of samples negative against H5 HA (Table 1) included the normal chicken serum, sera of SPF layer chickens and sera from commercial layers and broilers, which served as the nonvaccinated controls in the immunization studies with rH5-*E. coli*. Another category of anti-H5 HA negative samples was the reference antisera raised in the SPF chickens against the non-H5 subtype LPAIVs (Additional file 1: Table S3). These samples were positive against HAs of the H1–H4 and H6–H16 subtypes, as confirmed by the results of the HI assay with the respective virus strains.

**Fig. 1:**
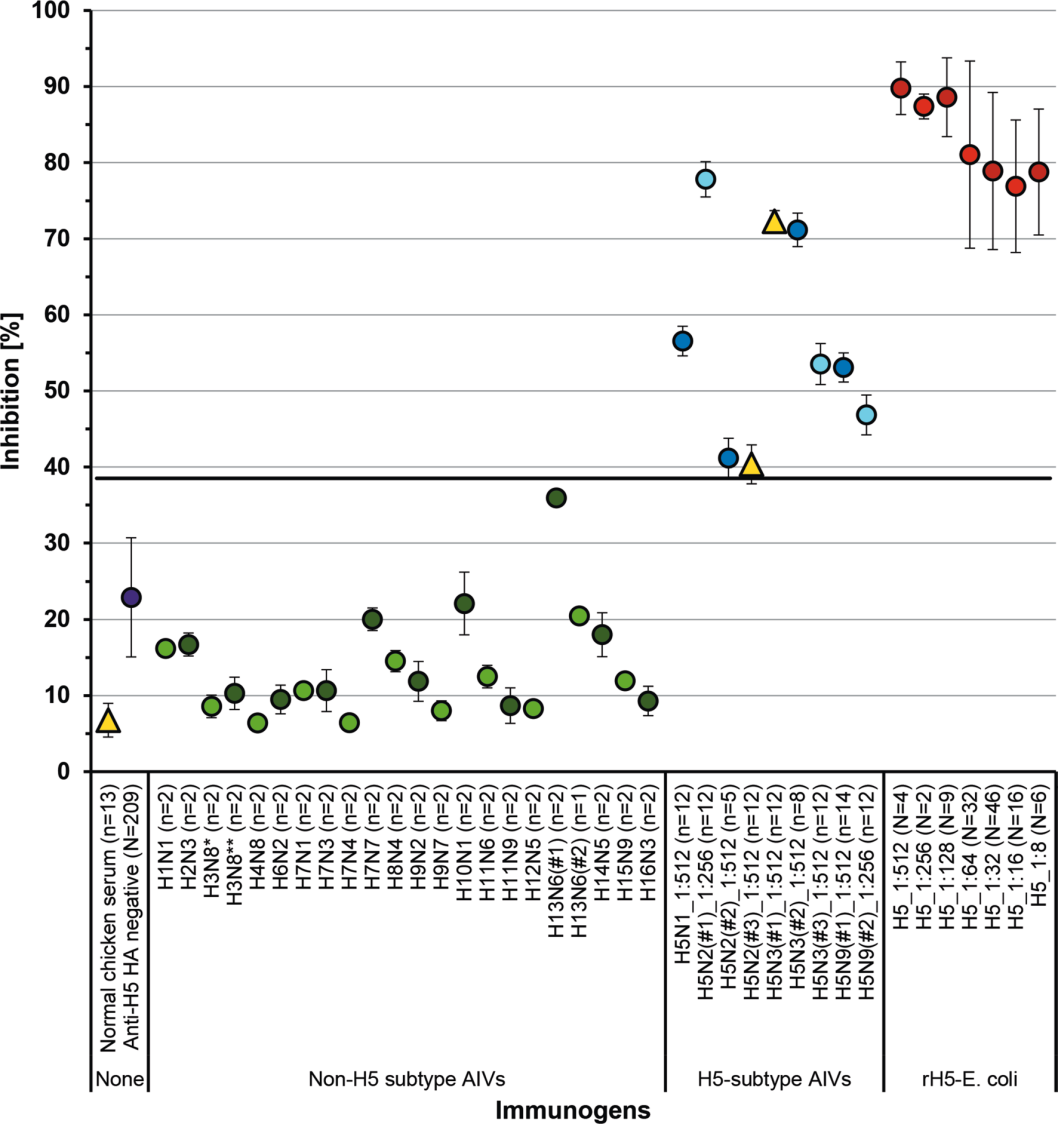
Discrimination between the anti-H5 HA positive and negative sera in the H5 EB-ELISA. Serum samples of the nonimmunized chickens and chickens immunized with LPAIVs and recombinant HA protein from the H5N1 HPAIV (rH5-*E. coli*) to obtain the reference and experimental antisera, respectively, were analyzed in the EB-ELISA as described in the Methods section. Data on the analyzed samples are provided in Table 1 and Additional file 1: Tables S2–S4. Annotations for the horizontal axes refer to serum category and subcategory. The reference antisera were denoted according to the HA and NA subtypes of AIV used for chicken inoculation. Denotations of antisera against H5-subtype AIVs were completed with HI titers determined using the homologous virus strains and provided in the certificates. The * and ** symbols were applied to discriminate between different virus strains of the same HA and NA subtype, while the # symbol followed by the numbers (1, 2 or 3) discriminate among the antiserum batches. Experimental antisera were denoted according to the vaccine HA subtype and HI titers against heterologous H5N2 LPAIV adapted from our previous paper [10]. Annotations of the vertical axes refer to the test results, expressed as the inhibition percentages. The cutoff value of the test was calculated from the inhibition percentages set for anti-H5 HA negative sera of the nonimmunized chickens (arithmetic mean + 2xSD) and shown as the horizontal line. The results for individual serum subcategories are presented as the arithmetic means ± SD of the inhibition percentages calculated for the indicated number of independent sample assays (n) or samples tested (N). Triangle and circle symbols represent the results for the control and the remaining sera, respectively. Samples showing inhibition above the cutoff value of 38.5% were considered positive against HA of H5-subtype influenza viruses

The chicken sera, for a total of 358 samples, were tested by H5 EB-ELISA to represent different sample categories, listed in Table 1, which were analyzed in parallel. Each assay included the mAb control, anti-H5N2 and anti-H5N3 LPAIV antisera as the weak and strong positive controls, respectively, and the normal chicken serum as a negative control (Table 1). The ability of individual specimens to block the binding of the G-7-27-18 mAb to rH5-BEVS was expressed in terms of inhibition percentage. The threshold for positivity and negativity in the H5 EB-ELISA, a cutoff value of 38.5%, was obtained by adding two standard deviation (SD) values to the arithmetic mean of the inhibition percentages set for 209 samples of various-origin sera that were negative against H5 HA. Data from analyses of the anti-H5 HA negative and positive sera (232 and 126 samples, respectively) were summarized by calculation of arithmetic means ± SD of the inhibition percentages determined for the independent assays of individual samples or samples in the serum groups. The first approach was applied to the normal chicken serum and anti-LPAIV reference antisera, while the second was applied to the anti-H5 HA negative sera of various origins and groups of anti-H5 HA positive experimental antisera with different HI titers. As shown in Fig. 1, the mean inhibition percentages for the anti-H5 HA negative sera from the nonimmunized chickens and chickens immunized with AIVs of the H1–H4 and H6–H16 subtypes were below the cutoff value of 38.5%. In contrast, the mean inhibition percentages for the anti-H5 HA positive reference and experimental antisera were above this value. Thus, the H5 EB-ELISA correctly discriminated between the anti-H5 HA negative sera, including those against the non-H5 subtype AIVs, and sera positive for antibodies against H5 HAs. The H5 subtype-specific antibodies were detected in the reference and experimental antisera despite the HA antigens used to obtain them having been from highly divergent H5-subtype influenza viruses. The HA1 subunits of HAs of the H5N3, H5N1, H5N9 and H5N2 LPAIVs and rH5-*E. coli* shared 90 to 100% of their amino acid sequence identities with the HA1 subunit of rH5-BEVS, which contained an epitope for detected serum antibodies and G-7-27-18 mAb (Additional file 1: Table S7).

The H5 EB-ELISA was characterized using the key criteria of assay validation, such as specificity, sensitivity and repeatability of the assay and HI test as the gold standard. Analytical and diagnostic specificities (Asp and Dsp, respectively) were evaluated by calculating the percentage of samples negative in this test among the sera lacking HI activity against H5-subtype AIVs (Table 1). Data obtained for sera of chickens immunized with LPAIVs of the H1–H4 and H6–H16 subtypes allowed us to establish the Asp value of 100% (Table 2). Based on the test results for sera from the nonimmunized chickens, the Dsp value of 97.6% was determined (Table 2).

**Table 2:** Preliminary validation of the H5 EB-ELISA

Validation criteria and the samples used	Samples [N]	Assays [n]	Results		
Analytical specificity (Asp)			True negative	False positive	Asp (%)
			TN	FP	TN/(TN + FP)
Reference antisera against non-H5 subtype LPAIVs	22	43	43	0	100
Diagnostic specificity (Dsp)			True negative	False positive	Dsp [%]
			TN	FP	TN/(TN + FP)
Various-origin sera negative against H5 HA	209	209	204	5^a^	97.6
Diagnostic sensitivity (Dse) 1			True positive	False negative	Dse 1 (%)
			TP	FN	TP/(TP + FN)
Reference antisera against H5-subtype LPAIVs	9	99	97	2^b^	98.0
Diagnostic sensitivity (Dse) 2			True positive	False negative	Dse 2 (%)
			TP	FN	TP/(TP + FN)
Experimental antisera against HA from H5N1 HPAIV	115	115	114	1^c^	99.1
Repeatability of assays			Mean [OD_450_]	SD^d^	RSD^e^ (%)
G-7-27-18 mAb control	1	13	1.566	0.111	7.1
Normal chicken serum (negative control)	1	13	1.460	0.114	7.8
Anti-H5N2 LPAIV antiserum (weak positive control)	1	12	0.944	0.083	8.8
Anti-H5N3 LPAIV antiserum (strong positive control)	1	12	0.439	0.044	10.0

Serum samples (Table 1) were classified as anti-H5 HA positive or negative based on the HI assay results. HI titers for the reference antisera were provided in the product certificates. Normal chicken serum and sera from commercial chickens immunized and nonimmunized with rH5-*E. coli* were analyzed in the HI assay according to the protocol included in the Methods section. Data for all experimental antisera and 96 out of 210 sera negative against H5 HA were adapted from our previous paper [10]. HI titers for the reference and experimental antisera against H5-subtype AIVs are provided in Table 1 and Additional file 1: Tables S2 and S4. In this study, serum HI titers equal to or greater than 1:8 were considered positive. The H5 EB-ELISA was performed, and inhibition percentages were calculated as described in the Methods section. Samples showing inhibition above the cutoff value of 38.5% were considered positive against HA of H5-subtype influenza viruses in the EB-ELISA. Analytical and diagnostic specificities were calculated by counting the samples determined in the EB-ELISA as true negatives among the anti-H5 HA negative sera from chickens immunized with LPAIVs of the H1–H4 and H6–H16 subtypes and the nonimmunized chickens, respectively. Diagnostic sensitivities 1 and 2 were calculated by counting the samples determined in the EB-ELISA as true positives among the anti-H5 HA positive reference and experimental antisera, respectively. The repeatability of the EB-ELISA was evaluated by performing the indicated number of assays for the listed control samples

^a^The samples yielding from 39.7 to 43.0% inhibition

^b^Two out of twelve assays of the anti-H5N2 LPAIV antiserum, batch #3, with an HI titer of 1:512

^c^The sample with an HI titer of 1:64

^d^Standard deviation

^e^Relative standard deviation

Diagnostic sensitivity (Dse) of the H5 EB-ELISA was evaluated by calculating the percentage of samples positive in the test among the antisera with HI titers against H5-subtype AIVs of 1:8 or greater (Table 1). In this way, Dse values of 98.0% and 99.1% were established from the assay results for the reference and experimental antisera, respectively (Table 2). Of note, the true positive sera included all of the samples with HI titers of 1:8 (Table 2), which would be interpreted as being negative according to recommendations of OIE [3]. In this context, the H5 EB-ELISA can be considered more sensitive than the HI assay in detecting H5 subtype-specific antibodies. The sensitivity of the H5 EB-ELISA was also assessed by a comparison to those of the commercial ELISA test, ID Screen Influenza H5 Antibody Competition-FluAC H5. The test is a diagnostic kit designed to specifically detect antibodies directed against the H5 antigen of influenza A viruses in bird sera. Analyses of the anti-H5 HA antisera using the FluAC H5 test were performed and interpreted as described in Additional file 4. According to the data in Additional file 4: Tables S1 and S2, the reference and experimental antisera were determined in the FluAC H5 test with 100% and 80.9% sensitivities, respectively. Thus, the developed H5 EB-ELISA was less sensitive than the commercial test in detecting the anti-H5 HA antibodies raised in chickens with H5-subtype influenza viruses. However, it was substantially more sensitive in testing the antisera obtained using the vaccine H5 HA protein for chicken immunization.

The high specificity and sensitivity of the H5 EB-ELISA, as indicated by values of Asp, Dsp and Dse (Table 2), were accompanied by a satisfactory repeatability of the assay. Values of relative standard deviation (RSD) from the independent testing of the mAb control, negative controls and positive controls were between 7.1 and 10.0% (Table 2). In summary, the developed H5 EB-ELISA fulfills criteria for effective detection of antibodies against HAs of H5-subtype influenza viruses.

## Discussion

The H5-subtype HPAIVs, such as the H5N1, H5N2, H5N6, and H5N8 viruses, are of both veterinary and public health concern worldwide. Infection with these viruses leads to multiorgan disease and death in domestic birds [5, 6]. After bird-to-human transmission, the H5N1 viruses cause severe disease with a frequently fatal outcomes, and their pandemic potential is commonly recognized [7]. For these reasons, H5-subtype HPAIVs and LPAIVs with a propensity to mutate into HP viruses are under epidemiologic surveillance as NAIVs, which is accomplished using a variety of methods recommended by OIE [3]. Our initial work in the area of H5Nx AIV diagnostics provided 6 different antibody clones against the HA1 subunit of HA [9]. Each of these mAbs showed broad strain specificity against AIVs of the H5 subtype and did not cross-react with non-H5 subtype virus strains. The study presented here resulted in a prototype H5 EB-ELISA for detecting anti-H5 HA antibodies in chicken sera, which was based on the G-7-27-18 mAb selected from the newly generated antibody clones.

Consistent with the characteristics of the blocking mAb [9], all of the analyzed antisera against HAs of the H1–H4 and H6–H16 subtypes tested negative in the H5 EB-ELISA (Fig. 1, Table 2), which proves that the assay is truly H5-subtype specific. In addition, the anti-H5 HA negative, various-origin sera were determined with high specificity (Fig. 1, Table 2). To further refine the H5 EB-ELISA, a larger number of anti-H5 HA negative specimens need to be analyzed to correct the cutoff value and/or indicate the doubtful threshold. Alternatively, the cutoff value could be optimized by using the receiver operating characteristic (ROC) analysis on the larger set of data.

The H5 EB-ELISA enabled not only specific but also sensitive detection of H5 subtype-specific antibodies, especially when applied to examine sera from chickens immunized using the H5 HA protein (Fig. 1, Table 2, Additional file 4: Table S2). Thus, the developed assay is particularly suitable for the differentiation of infected chickens from chickens vaccinated with anti-H5Nx virus vaccines based on recombinant H5 HA proteins. Most recently, the Volvac® B.E.S. T AI + ND vaccine containing the H5 HA protein of baculovirus-expression system origin has been positively evaluated by efficacy trials in commercial chickens [13]. In general, recombinant DNA technology has been widely explored to obtain influenza virus HA protein, as exemplified by rH5-*E. coli* [10, 12]. Thus, the contribution of such vaccines in anti-AIV vaccination programs can be expected to increase, similar to the utility of the H5 EB-ELISA with its current characteristics.

Our results confirmed that the H5 EB-ELISA performed well in screening the SPF chickens immunized with H5-subtype LPAIVs. Nevertheless, the sensitivity of detecting the anti-H5 HA antibodies in the reference antisera was lower than in the experimental antisera (Fig. 1, Table 2) and was decreased in comparison to those achieved with the FluAC H5 test (Additional file 4: Table S1). Therefore, it would be advisable to further optimize the H5 EB-ELISA to increase the sensitivity of detecting H5 subtype-specific serum antibodies induced with H5Nx influenza viruses. Thus, the optimized assay would be more effective as a DIVA test in vaccination programs utilizing predominantly oil-emulsified, inactivated whole AIV vaccines [4]. It would probably work better when used for the diagnosis of infection with H5-subtype influenza viruses.

Thus far, only a few experimental bELISA and cELISA tests designated to detect anti-H5 HA antibodies have been developed and evaluated [14–20]. These assays have employed mAbs predominantly to the highly variable HA1 subunit of HA [14–16, 20], and as an exception against the relatively well-conserved HA2 subunit of the antigen [17]. The tests reported by Chen et al. [14] and Yang et al. [15] did not show cross-reactions with non-H5 subtype AIVs; however, their confirmed H5-subtype specificities were restricted to a single H5N2 virus strain [14] or three strains representing the H5N1 and H5N2 AIVs [15]. Prabakaran et al. [16] developed bELISA based on the mAb to the linear epitope in the HA1 subunit with 100% and 96.9% conservation rates among the H5N1 virus isolates from humans and avian sources, respectively, while 54.3% conservation rates were observed in the H5-subtype viruses with an NA subtype other than N1. Consequently, the assay provided the highly sensitive detection of antibodies against HAs of various-origin H5N1 viruses in chicken and human sera, but predictably, it would not detect the anti-HA antibodies in approximately 50% of antisera raised against H5N2-N9 viruses. In contrast, Postel et al. [17] set up the broadly reacting cELISA for anti-H5 HA antibody detection using mAb, which recognized a linear epitope conserved within the H5-subtype influenza viruses and located in the HA2 subunit. The assay demonstrated good diagnostic specificity and sensitivity in testing sera from different avian species but also substantial cross-reactivity with H2 subtype-specific sera and, to a lesser extent, with sera specific for H1 and H6 subtypes. Thus, this H5 cELISA did not allow for a clear distinction between antisera against HAs of the H5 and H2, H1 or H6 subtypes. In the H5-subtype cELISA described by Dlugolenski et al. [18], no intersubtype cross-reactions were found, but the accuracy of the test was low and varied between the avian species tested. The diagnostic specificity and sensitivity of this assay calculated across all chicken, duck and turkey sera were 32% and 85%, respectively, and those for chicken sera were 63% and 66%, respectively. The high specific and sensitive detection of antibodies to H5 HA in chicken sera was achieved in the bELISA designed by Jensen et al. [19]. However, the high accuracy of the test was achieved only after two subsequent ELISAs were performed using the H5N7 and H5N2 inactivated viruses as the coating antigens to circumvent interference with the NA protein. In addition, the H5-subtype specificity of this bELISA was not adequately validated, as only five subtypes other than H5 were considered in the cross-reactivity testing.

The most promising studies presenting mAbs against H5 HA and the application of one of them, the 5D8 mAb, in the development of H5 cELISA were described by Moreno et al. [20]. The assay clearly differentiated homologous and heterologous positive sera and performed very well in terms of diagnostic specificity and sensitivity for different avian species. Such performance characteristics of the assay were due to the wide intra-H5-subtype reactivity of the 5D8 mAb. It was shown that the competitor antibody recognizes a conformational epitope in the receptor-binding domain of the HA1 subunit and inhibits hemagglutination by H5-subtype AIVs. This implies that the cELISA will not detect antibodies to influenza viruses mutated under selection pressure of HI antibodies targeting the binding epitope of the 5D8 mAb. Owing to usage of the G-7-27-18 mAb with no HI activity (Additional file 2: Tables S3 and S4), detectability of the H5-subtype-specific antibodies in the EB-ELISA presented here is not affected by mutations induced with HI antibodies. In this respect, the assay developed by us is advantageous over the cELISA reported by Moreno et al. [20].

The blocking antibody in the present H5 EB-ELISA combines the inability to inhibit hemagglutination with high and broad-range specificity against H5 HAs [9], indicating conservation of its target epitope within HAs of the H5-subtype influenza viruses. Thus, the assay can be expected to diagnose infections with the currently circulating and novel H5Nx viral strains. As such, the H5 EB-ELISA meets the demand for diagnostic tools to accurately identify the HA-subtype specificity of antibodies to AIV, which is reported despite the availability of commercial kits [4]. The examples are the FluAC H5 test (IDVet, France) and H5-HA antibody ELISA kit (Dialab, Germany), which are designed to detect the anti-H5 HA antibodies in bird and human sera, respectively. The diagnostic performance of the FluAC H5 test has been evaluated in the domestic poultry population of Vietnam, partially vaccinated with reassortant H5N1 LP virus vaccine [21], ducks experimentally infected with LP and HP H5-subtype AIVs or immunized with H5 HA-encoding DNA vaccine [22], waterfowl experimentally infected with LP and HP H5N1 AIVs [23], mute swans [24], and zoo birds vaccinated with inactivated H5N9 AI vaccine [25]. In some of these studies, the assay showed low degree of cross-reactivity with antisera to the non-H5 subtype AIVs [21], a specificity value of only 89.4% [22] and variable sensitivity depending on the tested viral strain [17, 23]. In particular, low rates of detecting anti-H5 HA antibodies, 14% and 22%, were noted for antisera against the A/Chicken/West Java/SMI-PAT/2006 H5N1 HPAIV [23] and Egyptian HPAIV H5N1 antigenic drift variant [17], respectively. To improve the performance of the FluAC H5 test, modifications to the manufacturer's protocols [23, 24] or revalidation of the cutoff value [21] are suggested. Evaluation of the H5-HA antibody ELISA kit showed that the assay detects only high levels of anti-H5 HA antibodies and may produce false positive results for antisera towards seasonal H3N2 and H1N1 influenza viruses [26].

The disadvantages of the referenced experimental and commercial ELISAs justify our efforts to provide a novel screening test for subtype-specific serodiagnosis and surveillance. The accuracy of the current H5 EB-ELISA is sufficiently high to consider its further optimization and validation in accordance with the OIE guidelines [27]. Future analyses should include sera from chickens infected with H5Nx viruses. Depending on the scope of validation work, the test could be used for serological analyses in chickens and other poultry species, such as geese, ducks or turkeys, as well as in wild birds. The present H5 EB-ELISA also provides a basis for developing an assay designed to diagnose infection with H5Nx influenza viruses in humans.

## Conclusions

The H5 EB-ELISA developed in this study performed well in terms of Asp, Dsp and Dse when applied to screen chicken sera for the presence of H5 subtype-specific antibodies. Thus, the assay warrants further optimization and validation work using a larger set of sera and ROC analysis to select the optimal cutoff, Dsp and Dse values. Furthermore, validation studies could be expanded with serological analyses in a variety of domestic and wild birds to provide a multispecies assay. In large-scale sero-surveillance examinations or AIV vaccination campaigns, an optimized and fully validated H5 EB-ELISA would be a useful alternative to the HI test. The current H5 EB-ELISA is also a valuable starting point to develop a diagnostic test for humans.

## Supplementary Information

**Additional file 1.** Supplementary data on antigens and sera. **Table S1.** Recombinant H5 HA proteins (ITC, OET Ltd., IBA). **Table S2.** Reference antisera against LPAIVs of the H5 subtype (x-OvO Ltd.). **Table S3.** Reference antisera against LPAIVs of the non-H5 subtypes (x-OvO Ltd.). **Table S4.** Experimental antisera against HA from H5N1 HPAIV (IBA). **Table S5.** Antigens and antisera (x-OvO Ltd.) used in the HI tests performed at IBA. **Table S6.** Homology of H5 HA antigens against rH5-mammalian, an immunogen used in mAb production. **Table S7.** Homology of H5 HA antigens against rH5-BEVS, the coating antigen in the EB-ELISA.

**Additional file 2.** Verification of mAb activity in the hemagglutination inhibition assay. **Table S1.** Antigens and antisera used in testing the mAbs for HI activity. **Table S2.** Commercial mAbs against H5 HA used as controls in the HI assays. **Table S3.** Results of the HI assay with H5N2 LPAIV. **Table S4.** Results of the HI assay with H5N3 LPAIV.

**Additional file 3.** Development and optimization of H5 EB-ELISA. **Fig. S1.** The ELISA titration curves of G-7-27-18 mAb against rH5-BEVS coated on the well surfaces with varied hydrophilicity. Titration curves were denoted according to the plate type. **Fig. S2.** The ELISA titration curves of G-7-27-18 mAb against rH5-BEVS under preliminary assay conditions. rH5-BEVS was coated at (A) 1.0 μg/mL or (B) 0.5 μg/mL on MediSorp plates. Titration curves were denoted according to the coating concentration of rH5-BEVS in μg per mL (1 μg or 0.5 μg) and then the time of plate incubation with G-7-27-18 mAb (60 min or 30 min), anti-mouse antibodies (60 min or 30 min) at the indicated dilution (1:1,000) and TMB (15 min or 10 min). **Fig. S3.** The ELISA titration curves of G-7-27-18 mAb against rH5-BEVS under assay conditions optimized in step 1. Titration curves were denoted according to the coating concentration of rH5-BEVS in μg per mL (0.5 μg) and then the time of plate incubation with G-7-27-18 mAb (60 min or 30 min), anti-mouse antibodies (60 min) at the indicated dilutions (1:1,000, 1:1,500, 1:2,000 or 1:2,500) and TMB (15 min). **Fig. S4.** The ELISA titration curves of G-7-27-18 mAb against rH5-BEVS under assay conditions optimized in step 2. Titration curves were denoted according to the coating concentration of rH5-BEVS in μg per mL (0.5 μg) and then the time of plate incubation with G-7-27-18 mAb (60 min or 30 min), anti-mouse antibodies (60 min) at the indicated dilutions (1:1,500, 1:2,000, 1:3,000 or 1:4,000) and TMB (15 min). **Fig. S5.** The ELISA titration curves of G-7-27-18 mAb against rH5-BEVS under assay conditions optimized in step 3. Titration curves were denoted according to the coating concentration of rH5-BEVS in μg per mL (0.5 μg) and then the time of plate incubation with G-7-27-18 mAb (60 min), anti-mouse antibodies (60 min) at the indicated dilutions (1:2,500, 1:3,000, 1:3,500 or 1:4,000) and TMB (15 min).

**Additional file 4.** Sensitivity of H5 EB-ELISA relative to the commercial FluAC H5 test. **Table S1.** Detection of H5 subtype-specific antibodies in the reference antisera. **Table S2.** Detection of H5 subtype-specific antibodies in the experimental antisera.

## Data Availability

The results described here are contained in the Patent U.S. 10,696,737 (2020-06-20) and Patent Applications P.418671 (2016-09-12) and PCT/PL2017/000084 (2017-09-11): Monoclonal antibodies against hemagglutinin of H5-subtype influenza viruses and uses thereof, hybridomas producing the said antibodies, compositions and diagnostic kits. The datasets supporting the conclusions of this article are included within the article and its additional files. The newly established G-7-27-18 hybridoma cell line producing G-7-27-18 mAb used in the presented EB-ELISA was given Accession Number DSM ACC3297 by the International Depositary Authority and is held by the Leibniz Institute DSMZ-German Collection of Microorganisms and Cell Cultures (Braunschweig, Germany).

## Abbreviations

IV: Influenza virus
RNA: Ribonucleic acid
NP: Nucleoprotein
M1: Matrix protein
M2: Membrane protein
NS1: Nonstructural protein
NEP: Nuclear export protein
HA: Hemagglutinin
NA: Neuraminidase
AIV: Avian influenza virus
LP: Low pathogenic
HP: Highly pathogenic
OIE: World Organization for Animal Health
NAIV: Notifiable avian influenza virus
rRT-PCR: Real-time reverse transcriptase polymerase chain reaction
AGID: Agar gel immunodiffusion
ELISA: Enzyme-linked immunosorbent assay
HI: Hemagglutination inhibition
NI: Neuraminidase inhibition
cELISA: Competitive enzyme-linked immunosorbent assay
bELISA: Blocking enzyme-linked immunosorbent assay
DIVA: Differentiation infected from vaccinated animals
MAb: Monoclonal antibody
EB: Epitope-blocking
IgG: Immunoglobulin G class
Fab: Antigen-binding fragment
Fc: Crystallizable fragment
MALDI-TOF/TOF: Matrix-Assisted Laser Desorption Ionization Time of Flight
SPF: Specific pathogen-free
BEVS: Baculovirus expression vector system
OD: Optical density
SD: Standard deviation
Asp: Analytical specificity
Dsp: Diagnostic specificity
Dse: Diagnostic sensitivity
RSD: Relative standard deviation
ROC: Receiver operating characteristic
