# Supplementary material for: A novel epitope-blocking ELISA for specific and sensitive detection of antibodies against H5-subtype influenza virus hemagglutinin
Source: Virol J. 2021 Apr 30;18:91. doi: 10.1186/s12985-021-01564-6 (PMC8085643; doi:10.1186/s12985-021-01564-6)
Supplement: Supplementary file 1 — Additional file 1. Supplementary data on antigens and sera. Table S1. Recombinant H5 HA proteins (ITC, OET Ltd., IBA). Table S2. Reference antisera against LPAIVs of the H5 subtype (x-OvO Ltd.). Table S3. Reference antisera against LPAIVs of the non-H5 subtypes (x-OvO Ltd.). Table S4. Experimental antisera against HA from H5N1 HPAIV (IBA). Table S5. Antigens and antisera (x-OvO Ltd.) used in the HI tests performed at IBA. Table S6. Homology of H5 HA antigens against rH5-mammalian, an immunogen used in mAb production. Table S7. Homology of H5 HA antigens against rH5-BEVS, the coating antigen in the EB-ELISA. [file 12985_2021_1564_MOESM1_ESM.pdf]

Additional file 1: Supplementary data on antigens and sera.

In the present study, recombinant hemagglutinin (HA) proteins and/or avian influenza viruses (AIVs) were used as antigens in serological tests or immunogens in the production of mAbs and antisera. HA antigens had sequences derived from a total of 27 virus strains, representing the highly pathogenic (HP) and low pathogenic (LP) phenotypes. Antisera against AIVs and recombinant HA protein were employed in the development of the epitope-blocking (EB) ELISA and/or its evaluation.

Recombinant H5 HA antigens, referred to as rH5-mammalian, rH5-BEVS, and rH5-*E. coli*, were produced in mammalian, baculoviral, and bacterial expression systems at the Immune Technology Corporation (ITC; New York, NY, USA), Oxford Expression Technologies Ltd. (OET Ltd.; Oxford, England, UK) and Institute of Biotechnology and Antibiotics (IBA; Warsaw, Poland), respectively. The viral antigens, LPAIVs, and anti-LPAIV antisera, certified by Istituto Zooprofilattico Sperimentale delle Venezie (IZSVe; Legnaro, Padova, Italy), were purchased from x-OvO Ltd. (Dunfermline, Scotland, UK). The reference antisera were produced in specific pathogen-free (SPF) chickens inoculated with LPAIVs of the H1–H16 subtypes.

Experimental antisera against HA from H5N1 HPAIV originated from immunization studies, described in our previous paper [Sączyńska V et al. Front Immunol. 2019;10:2006. doi: 10.3389/fimmu.2019.02006]. Commercial layer chickens were vaccinated twice at 4- and 6-week intervals with 25 µg, 15 µg, 10 µg, or 5 µg of rH5-*E. coli* and aluminum hydroxide (alum) adjuvant. Samples collected from the nonimmunized broilers and layers in these and unreported efficacy trials for rH5-*E. coli* were the source of the anti-H5 HA negative sera. The panel of samples negative against H5 HA also comprised sera of SPF chickens obtained from the National Veterinary Research Institute (Puławy, Poland) and normal chicken serum purchased from Abcam (Cambridge, England, UK).

Table S1. Recombinant H5 HA proteins (ITC, OET Ltd., IBA).

Name	HA protein	Relevant HPAIV strain	Application	Origin
rH5-mammalian	aa 17–530<br>ΔRRRKKR<br>6x His<br>~95% pure	A/Bar-headed Goose/Qinghai/12/05(H5N1)	Antigen in mouse immunization for monoclonal antibody production by the hybridoma method	ITC
rH5-BEVS	aa 17–530<br>ΔRRRKKR<br>6x His<br>~95% pure	A/swan/Poland/305-135V08/2006(H5N1)	Coating antigen in the EB-ELISA	OET Ltd.
rH5-*E. coli*	aa 17–522<br>ΔRRRKKR<br>75-80% pure	A/swan/Poland/305-135V08/2006(H5N1)	Vaccine antigen used to obtain experimental chicken antisera against H5 HA, analyzed in the HI assay and EB-ELISA	IBA

Table S2. Reference antisera against LPAIVs of the H5 subtype (x-OvO Ltd.).

Hemagglutinin	Avian influenza virus		Batch No.	HI titer[a]	Denotation in Table 1/Fig. 1
Subtype	Subtype	Strain	Preparation date		
H5	H5N1	A/mallard/Italy/3401/05(H5N1)	1/14<br>04/2014	1:512	#1/H5N1_1:512
	H5N2	A/turkey/Italy/80(H5N2)	1/10<br>02/2010	1:256	#1/H5N2(#1)_1:256
			1/11<br>05/2011	1:512	#2/H5N2(#2)_1:512
			1/12<br>03/2012	1:512	#3/H5N2(#3)_1:512
	H5N3	A/duck/Italy/775/04(H5N3)	1/13<br>01/2013	1:512	#1/H5N3(#1)_1:512
			1/06<br>07/2006	1:512	#2/H5N3(#2)_1:512
			1/12<br>09/2012	1:512	#3/H5N3(#3)_1:512
	H5N9	A/chicken/Italy/22A/98(H5N9)	2/08<br>07/2008	1:512	#1/H5N9(#1)_1:512
			1/12<br>02/2012	1:256	#2/H5N9(#2)_1:256

[a] According to the certificate.

Table S3. Reference antisera against LPAIVs of the non-H5 subtypes (x-OvO Ltd.).

Hemagglutinin	Avian influenza virus		Batch No.	HI titer[a]	Denotation in Table 1/Fig. 1
Subtype	Subtype	Strain	Preparation date		
H1	H1N1	A/duck/Italy/1447/05(H1N1)	3/11 12/2011	1:1024	H1N1
H2	H2N3	A/duck/Germany/1215/73(H2N3)	1/06 10/2006	1:256	H2N3
H3	H3N8	A/pass/Italy/6000/V00(H3N8)	2/07 12/2007	1:256	H3N8*
		A/psitt/Italy/2873/00(H3N8)	1/06 09/2006	1:256	H3N8**
H4	H4N8	A/cockatoo/England/72(H4N8)	2/07 02/2007	1:512	H4N8
H6	H6N2	A/turkey/Canada/65(H6N2)	1/06 04/2006	1:256	H6N2
H7	H7N1	A/chicken//Italy/1067/99(H7N1)	1/13 01/2013	1:1024	H7N1
	H7N3	A/turkey/Italy/9289/V02(H7N3)	1/12 01/2012	1:512	H7N3
	H7N4	A/mallard/Italy/4810-79/04(H7N4)	1/10 04/2010	1:512	H7N4
	H7N7	A/macaw/England/626/80(H7N7)	01/11 11/2011	1:512	H7N7
H8	H8N4	A/turkey/Ontario/6118/68(H8N4)	1/06 08/2006	1:512	H8N4
H9	H9N2	A/turkey/Wisconsin/66(H9N2)	1/12 03/2012	1:1024	H9N2
	H9N7	A/turkey/Scotland/1/70(H9N7)	2/07 04/2007	1:1024	H9N7
H10	H10N1	A/ostrich/South Africa/01(H10N1)	1/07 02/2007	1:512	H10N1
H11	H11N6	A/duck/England/56(H11N6)	1/06 11/2006	1:256	H11N6
	H11N9	A/duck/Memphis/546/174(H11N9)	1/07 02/2007	1:1024	H11N9
H12	H12N5	A/duck/Alberta/60/76(H12N5)	1/06 11/2006	1:128	H12N5
H13	H13N6	A/gull/Maryland/704/77(H13N6)[a]	1/06 01/2006	1:128	#1/H13N6(#1)
			1/07 09/2007	1:1024	#2/H13N6(#2)
H14	H14N5	A/mallard/Gurjev/263/82(H14N5)	1/06 08/2006	1:512	H14N5
H15	H15N9	A/shearwater/Australia/2576/79(H15N9)	1/07 06/2007	1:2048	H15N9
H16	H16N3	A/gull/Denmark/68110/02(H16N3)	1/06 12/2006	1:256	H16N3

[a] According to the certificate.

Table S4. Experimental antisera against HA from H5N1 HPAIV (IBA).

Group name[a]	Samples selected[b] [N]	Range of HI titers with H5N2 LPAIV[c]	Samples tested in the EB-ELISA			
			Group no.[d]	HI titer with H5N2 LPAIV[c]	Samples [N]	Denotation in Fig. 1
L-25/4	12	1:8–1:512	1	1:512	4	H5_1:512
L-15/4	11	1:8–1:64	2	1:256	2	H5_1:256
L-10/4	14	1:8–1:128	3	1:128	9	H5_1:128
L-5/4	10	1:8–64	4	1:64	32	H5_1:64
L-25/6	18	1:8–1:512	5	1:32	46	H5_1:32
L-15/6	18	1:16–1:128	6	1:16	16	H5_1:16
L-10/6	17	1:16–1:128	7	1:8	6	H5_1:8
L-5/6	15	1:16–1:512				

[a] According to the chicken type (L, layer), the dose of alum-adjuvanted rH5-*E. coli* in µg (25, 15, 10, and 5), and the time interval between doses in weeks (4 or 6).

[b] Samples were selected from HI-positive antisera collected 1 and/or 2 weeks after the boost.

[c] The results of the HI assay were adapted from Sączyńska V et al. [Front Immunol. 2019;10:2006. doi: 10.3389/fimmu.2019.02006].

[d] Samples were grouped according to their HI titers.

Table S5. Antigens and antisera (x-OvO Ltd.) used in the HI tests performed at IBA.

Name	Relevant LPAIV strain	Application
H5N2 LPAIV	A/turkey/Italy/80(H5N2)	Antigen
Anti-H5N2 LPAIV antiserum	A/turkey/Italy/80(H5N2)	Positive control
Anti-H7N4 LPAIV antiserum	A/mallard/Italy/4810-79/04(H7N4)	Negative control
Anti-H7N7 LPAIV antiserum	A/macaw/England/626/80(H7N7)	Negative control

Table S6. Homology of H5 HA antigens against rH5-mammalian, an immunogen used in mAb production.

Name	HA protein	Relevant AIV strain	HA1 subunit[a]	
			Max score[b]	aa seq identity[b]
rH5-mammalian	ectodomain (ΔRRRKKR,6x His)	A/Bar-headed Goose/Qinghai/12/05(H5N1)	679	100%
rH5-BEVS	ectodomain (ΔRRRKKR,6x His)	A/swan/Poland/305-135V08/2006(H5N1)	677	99%
rH5-*E. coli*	truncated ectodomain (ΔRRRKKR)	A/swan/Poland/305-135V08/2006(H5N1)	677	99%
H5N3 AIV	full-length	A/duck/Italy/775/04(H5N3)	636	93%
H5N1 AIV	full-length	A/mallard/Italy/3401/05(H5N1)	633	93%
H5N9 AIV	full-length	A/chicken/Italy/22A/98(H5N9)	619	90%
H5N2 AIV	full-length	A/turkey/Italy/80(H5N2)	615	90%

[a] Targeted by G-7-27-18 mAb and HI antibodies.

[b] Determined using the BLAST program hosted by NCBI by alignments against the 17–338-aa sequence of HA from the A/Bar-headed Goose/Qinghai/12/05(H5N1) HPAIV.

Table S7. Homology of H5 HA antigens against rH5-BEVS, the coating antigen in the EB-ELISA.

Name	HA protein	Relevant AIV strain	HA1 subunit[a]	
			Max score[b]	aa seq identity[b]
rH5-BEVS	ectodomain (ΔRRRKKR,6x His)	A/swan/Poland/305-135V08/2006(H5N1)	679	100%
rH5-mammalian	ectodomain (ΔRRRKKR,6x His)	A/Bar-headed Goose/Qinghai/12/05(H5N1)	677	99%
rH5-*E. coli*	truncated ectodomain (ΔRRRKKR)	A/swan/Poland/305-135V08/2006(H5N1)	679	100%
H5N3 AIV	full-length	A/duck/Italy/775/04(H5N3)	635	93%
H5N1 AIV	full-length	A/mallard/Italy/3401/05(H5N1)	632	93%
H5N9 AIV	full-length	A/chicken/Italy/22A/98(H5N9)	618	90%
H5N2 AIV	full-length	A/turkey/Italy/80(H5N2)	614	90%

[a] Targeted by G-7-27-18 and HI antibodies.

[b] Determined using the BLAST program hosted by NCBI by alignments against the 17–338-aa sequence of HA from the A/swan/Poland/305-135V08/2006(H5N1) HPAIV.
