# Supplementary material for: A novel epitope-blocking ELISA for specific and sensitive detection of antibodies against H5-subtype influenza virus hemagglutinin
Source: Virol J. 2021 Apr 30;18:91. doi: 10.1186/s12985-021-01564-6 (PMC8085643; doi:10.1186/s12985-021-01564-6)
Supplement: Supplementary file 2 — Additional file 2. Verification of mAb activity in the hemagglutination inhibition assay. Table S1. Antigens and antisera used in testing the mAbs for HI activity. Table S2. Commercial mAbs against H5 HA used as controls in the HI assays. Table S3. Results of the HI assay with H5N2 LPAIV. Table S4. Results of the HI assay with H5N3 LPAIV. [file 12985_2021_1564_MOESM2_ESM.pdf]

Additional file 2: Verification of mAb activity in the hemagglutination inhibition assay.

Monoclonal antibodies against H5 hemagglutinin (HA) of influenza viruses, denoted G-1-31-22, G-2-14-10, G-5-32-5, G-6-42-42, G-6-42-71, G-7-24-17 and G-7-27-18, were examined for their ability to inhibit hemagglutination by H5-subtype influenza viruses (IVs). The hemagglutination inhibition (HI) assay was performed using erythrocytes from specific pathogen-free (SPF) chickens (National Veterinary Research Institute, Puławy, Poland) and H5N2 and H5N3 low pathogenic avian IVs (LPAIVs) as antigens (Table S1). According to previously published results [Sączyńska V et al. Virol J. 2018;15:13. doi: 10.1186/s12985-017-0886-2], the H5N2 and H5N3 virus strains used in HI activity determination were recognized by G-1-31-22, G-2-14-10, G-5-32-5, G-6-42-42, G-6-42-71, G-7-24-17 and G-7-27-18 mAbs in the ELISA tests.

Each assay included control erythrocytes and control samples. The anti-H5N2 and anti-H5N3 LPAIV antisera were used as positive controls in the HI assays with H5N2 and H5N3 LPAIVs, respectively (Table S1). Additional controls in some HI assays were commercial mAbs against H5 HA specified as having HI activity (Table S2). In all assays, the anti-H7N7 LPAIV antiserum was used as a negative control (Table S1).

Assays were performed in V-bottom 96-well plates (CellStar/Greiner Bio-One, Frickenhausen, Germany). MAbs and control antisera were serially diluted two-fold in Dulbecco's PBS (Sigma-Aldrich, St Louis, MO, USA) and then incubated for 25 min with 4 hemagglutination units (HAU) of viral antigen, H5N2 or H5N3 LPAIV. Simultaneously, a 1:4 dilution of sample not containing the viral antigen was incubated to provide the erythrocyte control. Thereafter, a 1% erythrocyte suspension was added. The results were recorded after incubation for a minimum of 30 min. The HI titer was defined as the lowest mAb concentration or the reciprocal of the highest serum dilution that caused an inhibition of hemagglutination activity with 4 HAU of the inactivated antigen.

Table S1. Antigens and antisera used in testing the mAbs for HI activity.

Name	Relevant influenza virus strain	Application	Certification and origin
**HI assay with H5N2 influenza virus**			
H5N2 LPAIV	A/turkey/Italy/80(H5N2)	Antigen	Istituto Zooprofilattico Sperimentale delle Venezie (IZSVe; Legnaro, Padova, Italy)
Anti-H5N2 LPAIV antiserum	A/turkey/Italy/80(H5N2)	Positive control	
Anti-H7N7 LPAIV antiserum	A/macaw/England/626/80(H7N7)	Negative control	x-OvO Ltd. (Dunfermline, Scotland, UK)
**HI assay with H5N3 influenza virus**			
H5N3 LPAIV	A/duck/Italy/775/04(H5N3)	Antigen	Istituto Zooprofilattico Sperimentale delle Venezie (IZSVe; Legnaro, Padova, Italy)
Anti-H5N3 LPAIV antiserum	A/duck/Italy/775/04(H5N3)	Positive control	
Anti-H7N7 LPAIV antiserum	A/macaw/England/626/80(H7N7)	Negative control	x-OvO Ltd. (Dunfermline, Scotland, UK)

Table S2. Commercial mAbs against H5 HA used as controls in the HI assays.

Name	Denotation	Origin	Cat. No.
Monoclonal Antibody to Influenza A (Hemagglutinin H5) H5N1 – Purified	mAb 1	Acris Antibodies GmbH (Herford, Germany)	AM00941PU-N
Monoclonal Antibody to Influenza A (Hemagglutinin H5) H5N1 – Purified	mAb 2	Acris Antibodies GmbH (Herford, Germany)	AM00945PU-N
Influenza A H5 Antigen Antibody (15A6)	mAb 3	Pierce/Thermo Fisher Scientific (Waltham, MA, USA)	MA1-81928

Table S3. Results of the HI assay with H5N2 LPAIV.

MAb or control antiserum	MAb concentrations [μg/mL] Antiserum dilutions	Result	
		HI activity*	HI titer
G-1-31-22 mAb	0.5–526	−	n/a
G-2-14-10 mAb	1.0–1011	−	n/a
G-5-32-5 mAb	1.0–1008	−	n/a
G-6-42-42 mAb	1.0–1034	−	n/a
G-6-42-71 mAb	0.9–973	−	n/a
G-7-24-17 mAb	0.9–877	−	n/a
G-7-27-18 mAb	1.0–1023	−	n/a
Anti-H5N2 LPAIV antiserum	1:4–1:4096	+	1:512
Anti-H7N7 LPAIV antiserum	1:4–1:4096	−	n/a

* Positivity and negativity in the assay are indicated by plus and minus symbols, respectively.

Table S4. Results of the HI assay with H5N3 LPAIV.

MAb or control antiserum	MAb concentrations [μg/mL] Antiserum dilutions	Result	
		HI activity*	HI titer
G-1-31-22 mAb	0.3–526	−	n/a
G-2-14-10 mAb	0.8–1011	−	n/a
G-5-32-5 mAb	0.9–1008	−	n/a
G-6-42-42 mAb	0.7–1034	−	n/a
G-6-42-71 mAb	0.9–973	−	n/a
G-7-24-17 mAb	0.5–877	−	n/a
G-7-27-18 mAb	0.9–1023	−	n/a
Mixture of G-1-31-22, G-2-14-10, G-5-32-5, G-6-42-42, G-6-42-71, G-7-24-17 and G-7-27-18 mAbs	0.7–726	−	n/a
mAb 1	0.1–125	+	1.0 μg/mL
mAb 2	0.1–125	+	2.0 μg/mL
mAb 3	0.1–125	+	7.8 μg/mL
Anti-H5N3 LPAIV antiserum	1:4–1:2048	+	1:512
Anti-H7N7 LPAIV antiserum	1:4–1:2048	−	n/a

* Positivity and negativity in the assay are indicated by plus and minus symbols, respectively.
