# Supplementary material for: A novel epitope-blocking ELISA for specific and sensitive detection of antibodies against H5-subtype influenza virus hemagglutinin
Source: Virol J. 2021 Apr 30;18:91. doi: 10.1186/s12985-021-01564-6 (PMC8085643; doi:10.1186/s12985-021-01564-6)
Supplement: Supplementary file 3 — Additional file 3. Development and optimization of H5 EB-ELISA. Fig. S1. The ELISA titration curves of G-7-27-18 mAb against rH5-BEVS coated on the well surfaces with varied hydrophilicity. Titration curves were denoted according to the plate type. Fig. S2. The ELISA titration curves of G-7-27-18 mAb against rH5-BEVS under preliminary assay conditions. rH5-BEVS was coated at (A) 1.0 μg/mL or (B) 0.5 μg/mL on MediSorp plates. Titration curves were denoted according to the coating concentration of rH5-BEVS in μg per mL (1 μg or 0.5 μg) and then the time of plate incubation with G-7-27-18 mAb (60 min or 30 min), anti-mouse antibodies (60 min or 30 min) at the indicated dilution (1:1,000) and TMB (15 min or 10 min). Fig. S3. The ELISA titration curves of G-7-27-18 mAb against rH5-BEVS under assay conditions optimized in step 1. Titration curves were denoted according to the coating concentration of rH5-BEVS in μg per mL (0.5 μg) and then the time of plate incubation with G-7-27-18 mAb (60 min or 30 min), anti-mouse antibodies (60 min) at the indicated dilutions (1:1,000, 1:1,500, 1:2,000 or 1:2,500) and TMB (15 min). Fig. S4. The ELISA titration curves of G-7-27-18 mAb against rH5-BEVS under assay conditions optimized in step 2. Titration curves were denoted according to the coating concentration of rH5-BEVS in μg per mL (0.5 μg) and then the time of plate incubation with G-7-27-18 mAb (60 min or 30 min), anti-mouse antibodies (60 min) at the indicated dilutions (1:1,500, 1:2,000, 1:3,000 or 1:4,000) and TMB (15 min). Fig. S5. The ELISA titration curves of G-7-27-18 mAb against rH5-BEVS under assay conditions optimized in step 3. Titration curves were denoted according to the coating concentration of rH5-BEVS in μg per mL (0.5 μg) and then the time of plate incubation with G-7-27-18 mAb (60 min), anti-mouse antibodies (60 min) at the indicated dilutions (1:2,500, 1:3,000, 1:3,500 or 1:4,000) and TMB (15 min). [file 12985_2021_1564_MOESM3_ESM.pdf]

# Additional file 3: Development and optimization of H5 EB-ELISA.

An epitope-blocking (EB) ELISA was developed using the purified recombinant H5 HA protein (aa 17–530, ΔRRRKKR, 6x His) produced in a baculovirus-expression vector system (BEVS; Oxford Expression Technologies Ltd.). Details of the protein, referred to as rH5-BEVS, are provided in Additional file 1: Table S1. During the assay development, the optimal type of ELISA plate and preliminary testing conditions were established. Further optimization of H5 EB-ELISA was performed in three steps.

## Plate selection

The PolySorp, MediSorp, MaxiSorp and MultiSorp plates (Nunc, Roskilde, Denmark) were coated by overnight incubation at 2–8 °C with 50 µL/well of rH5-BEVS at a concentration of 1.0 µg/mL in PBS. The coated plates were washed three times with 300 µL/well of PBS containing 0.05% Tween 20 (PBST; pH 7.4) and then incubated with 200 µL/well of 1% BSA in PBS for 60 min at room temperature (23 ± 2 °C). After blocking, the plates were washed two times with 350 µL/well of PBST. Next, 50 µL/well of G-7-27-18 mAb, two-fold serially diluted from 8,000 ng/mL to 0.977 ng/mL in 1% BSA in PBS, was applied to the plates, which were then incubated for 30 min at 37 °C and washed three times with 300 µL/well of PBST afterwards.

Antigen-bound mAbs were detected using HRP-labeled, anti-mouse IgG (γ-chain specific) antibodies (cat. no. A3673, lot no. 051M6270, Sigma-Aldrich). The plates were incubated with 50 µL/well of anti-mouse antibodies, diluted 1:1,000 in 1% BSA in PBS, for 30 min at 37 °C and then washed three times with 300 µL/well of PBST. The reactions were developed with 50 µL/well of TMB (Sigma-Aldrich) at room temperature (23 ± 2 °C) in the dark for 10 min and subsequently stopped by adding 50 µL/well of 0.5 M $H_2SO_4$. The optical density (OD) was measured at 450 nm ($OD_{450}$) using a Synergy 2 multidetection microplate reader (BioTek Instruments Inc., Winooski, VT, USA).

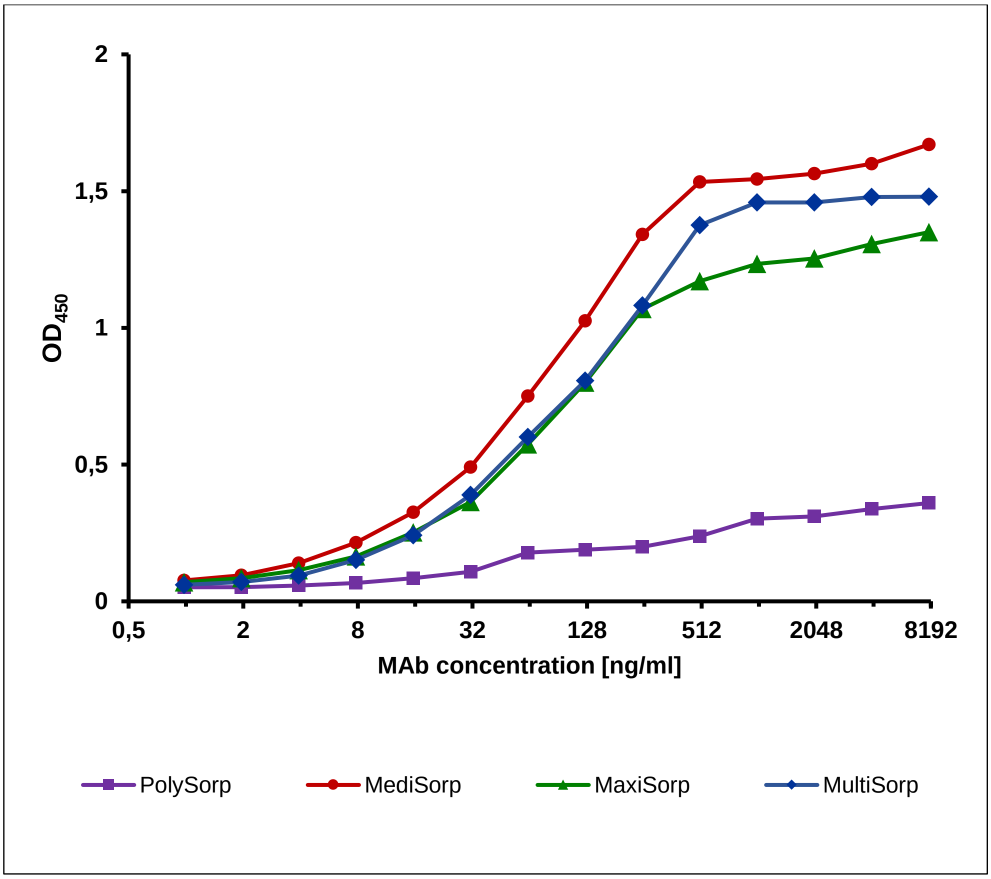


**Fig. S1. The ELISA titration curves of G-7-27-18 mAb against rH5-BEVS coated on the well surfaces with varied hydrophilicity.**
Titration curves were denoted according to the plate type.

## Preliminary assay conditions

The MediSorp plates (Nunc, Roskilde, Denmark) were coated by overnight incubation at 2–8 °C with 50 µL/well of rH5-BEVS at concentrations of 1.0 µg/mL or 0.5 µg/mL in PBS. The coated plates were washed three times with 300 µL/well of PBS containing 0.05% Tween 20 (PBST; pH 7.4) and then incubated with 200 µL/well of 1% BSA in PBS for 60 min at room temperature (23 ± 2 °C). After blocking, the plates were washed two times with 350 µL/well of PBST. Next, 50 µL/well of G-7-27-18 mAb, two-fold serially diluted from 2,000 ng/mL to 15.625 ng/mL in 1% BSA in PBS, was applied to the plates, which were then incubated for 60 min or 30 min at 37 °C and washed three times with 300 µL/well of PBST afterwards.

Antigen-bound mAbs were detected using HRP-labeled, anti-mouse IgG (γ-chain specific) antibodies (cat. no. A3673, lot no. 051M6270, Sigma-Aldrich). The plates were incubated with 50 µL/well of anti-mouse antibodies, diluted 1:1,000 in 1% BSA in PBS, for 60 min or 30 min at 37 °C and then washed three times with 300 µL/well of PBST. The reactions were developed with 50 µL/well of TMB (Sigma-Aldrich) at room temperature (23 ± 2 °C) in the dark for 15 min and/or 10 min and subsequently stopped by adding 50 µL/well of 0.5 M $H_2SO_4$. The optical density (OD) was measured at 450 nm ($OD_{450}$) using a Synergy 2 multidetection microplate reader (BioTek Instruments Inc., Winooski, VT, USA).

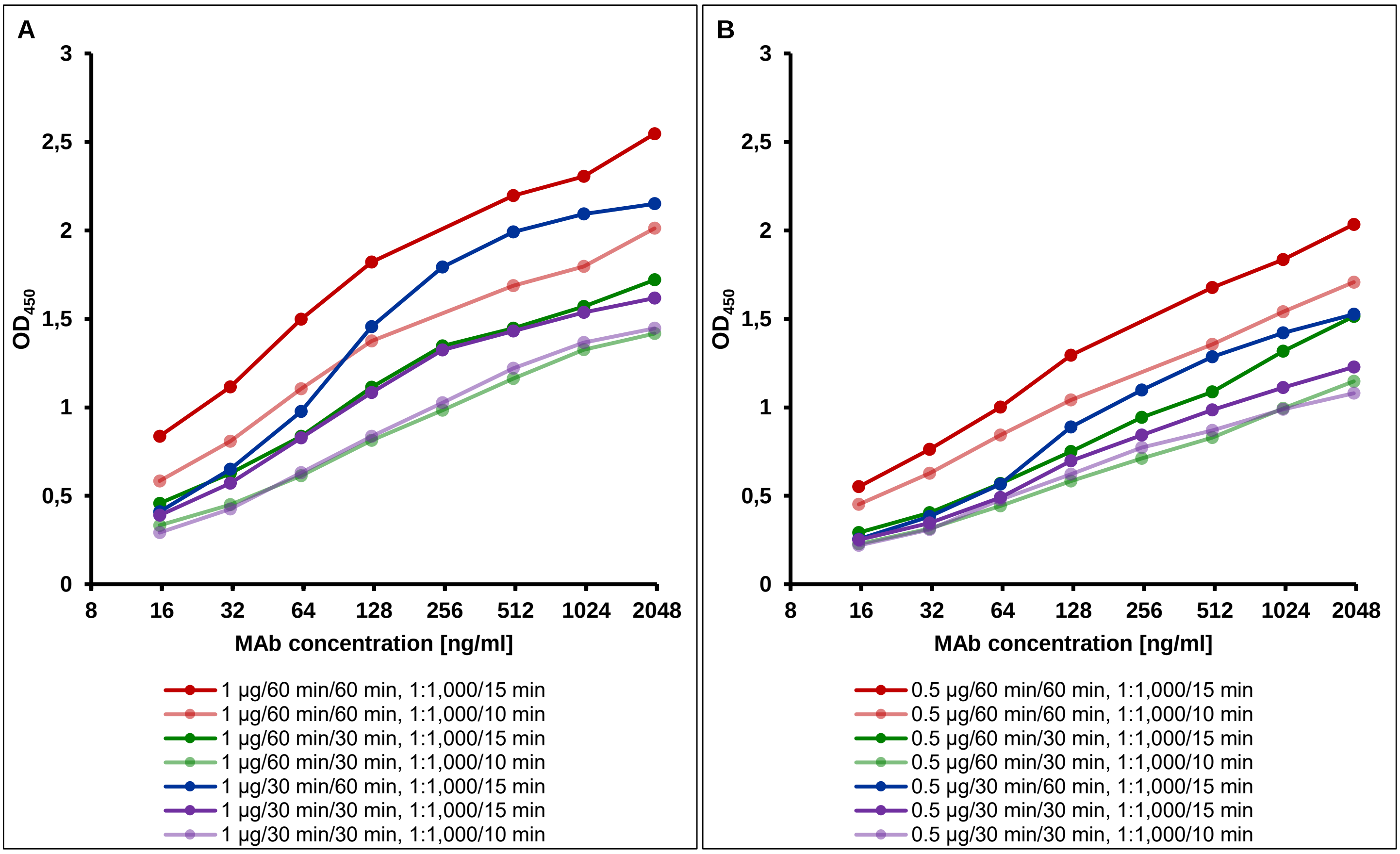


**Fig. S2. The ELISA titration curves of G-7-27-18 mAb against rH5-BEVS under preliminary assay conditions.**

rH5-BEVS was coated at (A) 1.0 µg/mL or (B) 0.5 µg/mL on MediSorp plates. Titration curves were denoted according to the coating concentration of rH5-BEVS in µg per mL (1 µg or 0.5 µg) and then the time of plate incubation with G-7-27-18 mAb (60 min or 30 min), anti-mouse antibodies (60 min or 30 min) at the indicated dilution (1:1,000) and TMB (15 min or 10 min).

## Optimization - step 1

The MediSorp plates (Nunc, Roskilde, Denmark) were coated by overnight incubation at 2–8 °C with 50 µL/well of rH5-BEVS at a concentration of 0.5 µg/mL in PBS. The coated plates were washed three times with 300 µL/well of PBS containing 0.05% Tween 20 (PBST; pH 7.4) and then incubated with 200 µL/well of Protein-Free T20 (PBS) Blocking Buffer (Pierce/Thermo Fisher Scientific) for 60 min at room temperature (23 ± 2 °C). After blocking, the plates were washed two times with 350 µL/well of PBST and then incubated with 100 µL/well of 1% BSA in PBS for 60 min at 37 °C with shaking at 150 rpm and subsequently washed three times with 300 µL/well of PBST. Next, 50 µL/well of G-7-27-18 mAb, two-fold serially diluted from 4,000 ng/mL to 3.906 ng/mL in 1% BSA in PBS, was applied to the plates, which were then incubated for 60 min or 30 min at 37 °C with shaking at 150 rpm and washed three times with 300 µL/well of PBST afterwards.

Antigen-bound mAbs were detected using HRP-labeled, anti-mouse IgG (γ-chain specific) antibodies (cat. no. A3673, lot no. 051M6270, Sigma-Aldrich). The plates were incubated with 50 µL/well of anti-mouse antibodies, diluted 1:1,000, 1:1,500, 1:2,000 or 1:2,500 in 1% BSA in PBS, for 60 min at 37 °C with shaking at 150 rpm and then washed three times with 300 µL/well of PBST. The reactions were developed with 50 µL/well of TMB (Sigma-Aldrich) at room temperature (23 ± 2 °C) in the dark for 15 min and subsequently stopped by adding 50 µL/well of 0.5 M $H_2SO_4$. The optical density (OD) was measured at 450 nm ($OD_{450}$) using a Synergy 2 multidetection microplate reader (BioTek Instruments Inc., Winooski, VT, USA).

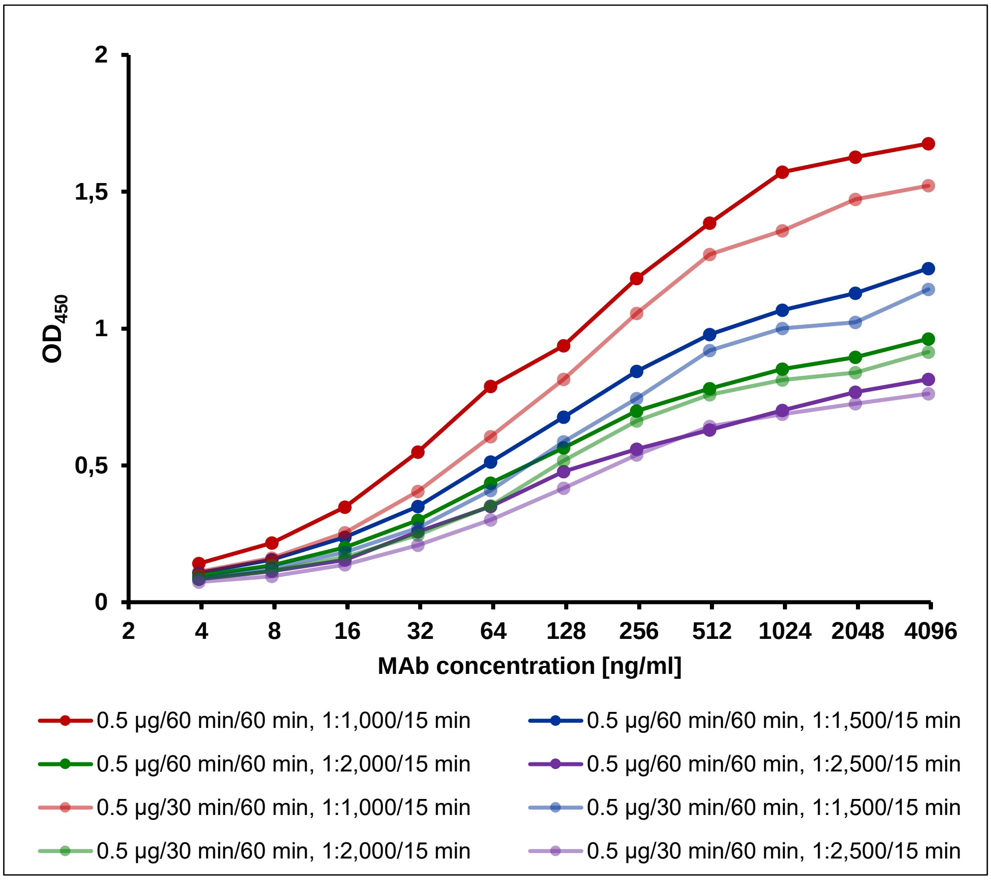


**Fig. S3. The ELISA titration curves of G-7-27-18 mAb against rH5-BEVS under assay conditions optimized in step 1.**

Titration curves were denoted according to the coating concentration of rH5-BEVS in µg per mL (0.5 µg) and then the time of plate incubation with G-7-27-18 mAb (60 min or 30 min), anti-mouse antibodies (60 min) at the indicated dilutions (1:1,000, 1:1,500, 1:2,000 or 1:2,500) and TMB (15 min).

## Optimization - step 2

The MediSorp plates (Nunc, Roskilde, Denmark) were coated by overnight incubation at 2–8 °C with 50 µL/well of rH5-BEVS at a concentration of 0.5 µg/mL in PBS. The coated plates were washed three times with 300 µL/well of PBS containing 0.05% Tween 20 (PBST; pH 7.4) and then incubated with 200 µL/well of Protein-Free T20 (PBS) Blocking Buffer (Pierce/Thermo Fisher Scientific) for 60 min at room temperature (23 ± 2 °C). After blocking, the plates were washed two times with 350 µL/well of PBST and then incubated with 100 µL/well of 1% BSA in PBS for 60 min at 37 °C with shaking at 150 rpm and subsequently washed three times with 300 µL/well of PBST. Next, 50 µL/well of G-7-27-18 mAb, two-fold serially diluted from 4,000 ng/mL to 3.906 ng/mL in 1% BSA in PBS, was applied to the plates, which were then incubated for 60 min or 30 min at 37 °C with shaking at 150 rpm and washed three times with 300 µL/well of PBST afterwards.

Antigen-bound mAbs were detected using HRP-labeled, anti-mouse IgG (γ-chain specific) antibodies (cat. no. A3673, lot no. SLBH0520, Sigma-Aldrich). The plates were incubated with 50 µL/well of anti-mouse antibodies, diluted 1:1,500, 1:2,000, 1:3,000 or 1:4,000 in HRP-Protector (CANDOR Bioscience GmbH), for 60 min at 37 °C with shaking at 150 rpm and then washed three times with 300 µL/well of PBST. The reactions were developed with 50 µL/well of TMB (Sigma-Aldrich) at room temperature (23 ± 2 °C) in the dark for 15 min and subsequently stopped by adding 50 µL/well of 0.5 M $H_2SO_4$. The optical density (OD) was measured at 450 nm ($OD_{450}$) using a Synergy 2 multidetection microplate reader (BioTek Instruments Inc., Winooski, VT, USA).

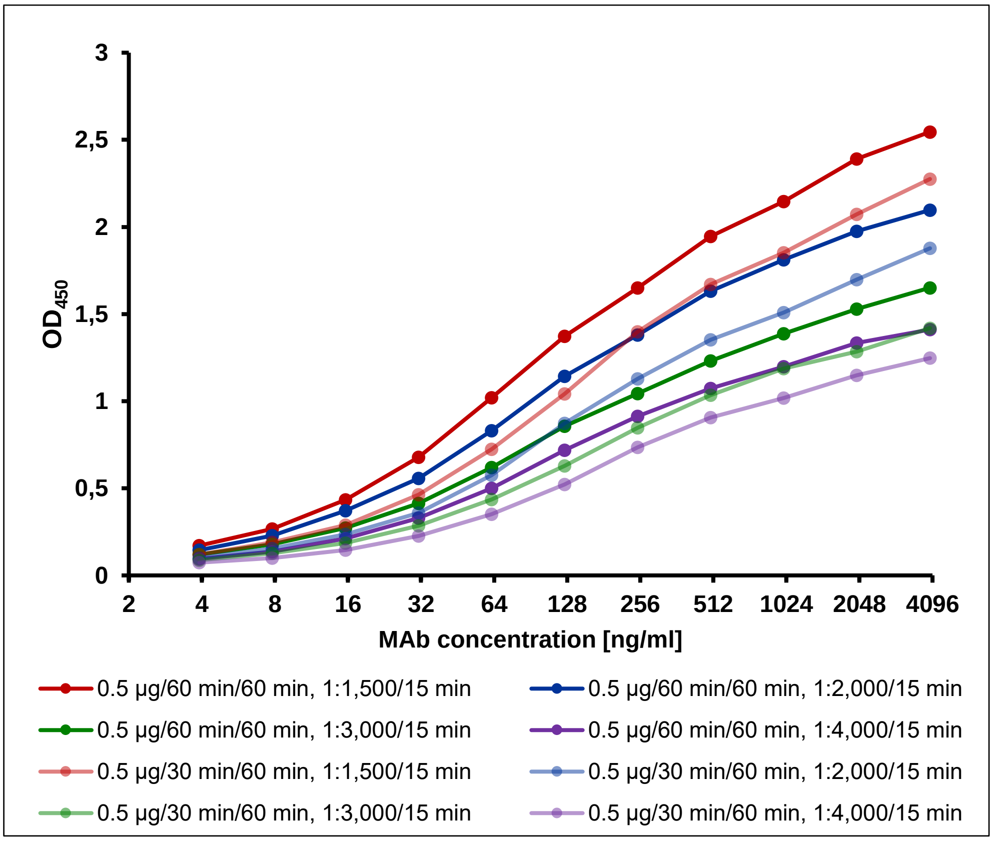


**Fig. S4. The ELISA titration curves of G-7-27-18 mAb against rH5-BEVS under assay conditions optimized in step 2.**

Titration curves were denoted according to the coating concentration of rH5-BEVS in µg per mL (0.5 µg) and then the time of plate incubation with G-7-27-18 mAb (60 min or 30 min), anti-mouse antibodies (60 min) at the indicated dilutions (1:1,500, 1:2,000, 1:3,000 or 1:4,000) and TMB (15 min).

## Optimization - step 3

The MediSorp plates (Nunc, Roskilde, Denmark) were coated by overnight incubation at 2–8 °C with 50 µL/well of rH5-BEVS at a concentration of 0.5 µg/mL in PBS. The coated plates were washed three times with 300 µL/well of PBS containing 0.05% Tween 20 (PBST; pH 7.4) and then incubated with 200 µL/well of Protein-Free T20 (PBS) Blocking Buffer (Pierce/Thermo Fisher Scientific) for 60 min at room temperature (23 ± 2 °C). After blocking, the plates were washed two times with 350 µL/well of PBST and then incubated with 100 µL/well of 1% BSA in PBS for 60 min at 37 °C with shaking at 150 rpm and subsequently washed three times with 300 µL/well of PBST. Next, 50 µL/well of G-7-27-18 mAb, two-fold serially diluted from 4,000 ng/mL to 3.906 ng/mL in Antibody Stabilizer PBS (CANDOR Bioscience GmbH, Wangen, Germany), was applied to the plates, which were then incubated for 60 min at 37 °C with shaking at 150 rpm and washed three times with 300 µL/well of PBST afterwards.

Antigen-bound mAbs were detected using HRP-labeled, anti-mouse IgG (γ-chain specific) antibodies (cat. no. A3673, lot no. SLBH0520, Sigma-Aldrich). The plates were incubated with 50 µL/well of anti-mouse antibodies, diluted 1:2,500, 1:3,000, 1:3,500 or 1:4,000 in HRP-Protector (CANDOR Bioscience GmbH), for 60 min at 37 °C with shaking at 150 rpm and then washed three times with 300 µL/well of PBST. The reactions were developed with 50 µL/well of TMB (Sigma-Aldrich) at room temperature (25 ± 0.1 °C) in the dark for 15 min and subsequently stopped by adding 50 µL/well of 0.5 M $H_2SO_4$. The optical density (OD) was measured at 450 nm ($OD_{450}$) using a Synergy 2 multidetection microplate reader (BioTek Instruments Inc., Winooski, VT, USA).

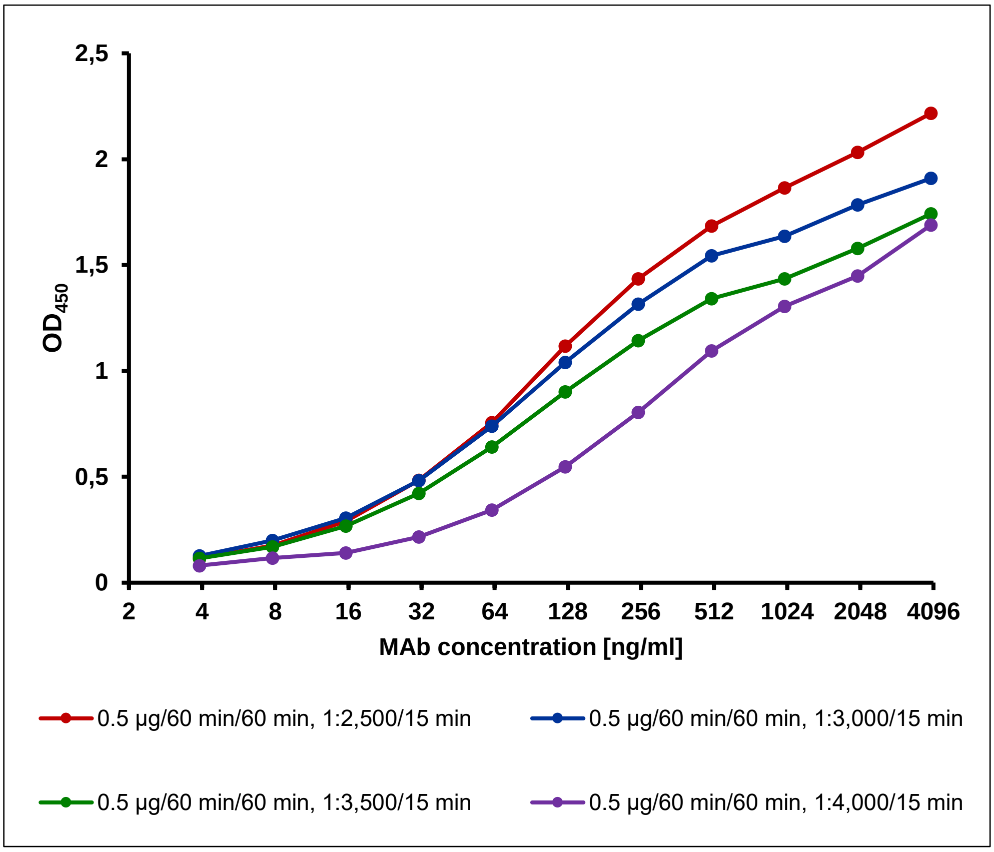


**Fig. S5. The ELISA titration curves of G-7-27-18 mAb against rH5-BEVS under assay conditions optimized in step 3.**

Titration curves were denoted according to the coating concentration of rH5-BEVS in µg per mL (0.5 µg) and then the time of plate incubation with G-7-27-18 mAb (60 min), anti-mouse antibodies (60 min) at the indicated dilutions (1:2,500, 1:3,000, 1:3,500 or 1:4,000) and TMB (15 min).
