# Supplementary material for: A novel epitope-blocking ELISA for specific and sensitive detection of antibodies against H5-subtype influenza virus hemagglutinin
Source: Virol J. 2021 Apr 30;18:91. doi: 10.1186/s12985-021-01564-6 (PMC8085643; doi:10.1186/s12985-021-01564-6)
Supplement: Supplementary file 4 — Additional file 4. Sensitivity of H5 EB-ELISA relative to the commercial FluAC H5 test. Table S1. Detection of H5 subtype-specific antibodies in the reference antisera. Table S2. Detection of H5 subtype-specific antibodies in the experimental antisera. [file 12985_2021_1564_MOESM4_ESM.pdf]

Additional file 4: Sensitivity of H5 EB-ELISA relative to the commercial FluAC H5 test.

Evaluation of the newly developed H5 EB-ELISA was expanded with comparison of its performance to the commercial ELISA test, ID Screen Influenza H5 Antibody Competition - FluAC H5 (IDVet, Grables, France). To this aim, the reference chicken antisera against H5N1, H5N2, H5N3 and H5N9 LPAIVs (x-OvO Ltd., Dunfermline, Scotland, UK), predetermined in the EB-ELISA, were subjected to examination in the FluAC H5 test. Here, we also exploited results from serological analyses of samples collected during immunization studies, described previously [Sączyńska V et al. Front Immunol. 2019;10:2006. doi: 10.3389/fimmu.2019.02006]. Commercial layer chickens were vaccinated twice at 4- and 6-week intervals with 25 µg, 15 µg, 10 µg, or 5 µg of H5 hemagglutinin (HA) protein produced in bacteria (rH5-*E. coli*) in the presence of aluminum hydroxide adjuvant. Postvaccination sera were analyzed for anti-H5 HA antibodies using hemagglutination inhibition (HI), indirect ELISA and FluAC H5 tests.

The reference and experimental antisera were classified as anti-H5 HA positive based on the results of the HI assay with homologous LPAIVs and heterologous H5N2 LPAIV, respectively. The HI titers for reference antisera were provided in the product certificates. The HI assay for experimental antisera was performed as described in the Methods section. In this study, serum HI titers equal to or greater than 1:8 were considered positive.

The H5 EB-ELISA was performed, and inhibition percentages were calculated as described in the Methods section. Samples showing inhibition above the cutoff value of 38.5% were considered positive against HA of the H5-subtype influenza viruses. The FluAC H5 test was performed according to the manufacturer's instructions. All serum samples were analyzed following the protocol, which improves the detection and sensitivity of the test and is suitable for chickens. The sample/negative control absorbance ratio of test samples was calculated and expressed as a competition percentage. Samples presenting a competition percentage ≥ 40%, between 35% and 40%, or ≤ 35% were considered negative, doubtful, or positive for the presence of anti-H5 HA antibodies, respectively.

Table S1. Detection of H5 subtype-specific antibodies in the reference antisera.

Antiserum[a]	Batch number[a]	HI titer with homologous LPAIVs[b]	Results in H5 EB-ELISA			Results in FluAC H5 test		
			Assays [n]	True positive TP	False negative FN	Assays [n]	True positive TP	False negative FN
Anti-H5N1 LPAIV	#1	1:512	12	12	0	1	1	0
Anti-H5N2 LPAIV	#1	1:256	12	12	0	1	1	0
	#2	1:512	5	5	0	1	1	0
	#3	1:512	12	10	2	1	1	0
Anti-H5N3 LPAIV	#1	1:512	12	12	0	1	1	0
	#3	1:512	12	12	0	1	1	0
Anti-H5N9 LPAIV	#1	1:512	14	14	0	1	1	0
	#2	1:256	12	12	0	1	1	0
Total	8 samples	1:256 or 1:512	91	89	2	8	8	0
**Diagnostic sensitivity 1[c]**		**TP/(TP+FN)**	**Dse 1 [%]**		**97.8**	**Dse 1 [%]**		**100**

[a] Certified by Istituto Zooprofilattico Sperimentale delle Venezie (IZSVe; Legnaro, Padova, Italy). Details of reference antisera against H5-subtype AIVs are provided in Additional file 1: Table S2.

[b] According to the certificate.

[c] Diagnostic sensitivities of EB-ELISA and FluAC test were calculated by counting the samples determined as true positives among the reference antisera that were classified as anti-H5 HA positive based on the HI assay results.

Table S2. Detection of H5 subtype-specific antibodies in the experimental antisera.

Group number[a]	HI titer with H5N2 LPAIV[b]	Samples [N]	Results in H5 EB-ELISA		Results in FluAC H5 test[b]	
			True positive TP	False negative FN	True positive TP	False negative FN
1	1:512	4	4	0	4	0
2	1:256	2	2	0	2	0
3	1:128	9	9	0	6	3
4	1:64	32	31	1	29	3
5	1:32	46	46	0	35	11
6	1:16	16	16	0	12	4
7	1:8[c]	6	6	0	5	1
Total	1:8–1:512	115	114	1	93	22
**Diagnostic sensitivity 2[d]**		**TP/(TP+FN)**	**Dse 2 [%]**	**99.1**	**Dse 2 [%]**	**80.9**

[a] Samples were selected from HI-positive antisera collected 1 and/or 2 weeks after the boost and grouped according to their HI titers. Details of the experimental antisera are provided in Additional file 1: Table S4.

[b] Data were adapted from Sączyńska V et al. [Front Immunol. 2019;10:2006. doi: 10.3389/fimmu.2019.02006].

[c] Not considered HI-positive according to the World Organization for Animal Health (OIE) Manual of Diagnostic Tests and Vaccines for Terrestrial Animals 2018. Chapter 3.3.4. Avian influenza (infection with avian influenza viruses). Available: https://www.oie.int/fileadmin/Home/eng/Health_standards/tahm/3.03.04_AI.pdf.

[d] Diagnostic sensitivities of EB-ELISA and FluAC test were calculated by counting the samples determined as true positives among the experimental antisera that were classified as anti-H5 HA positive based on the HI assay results.
